# How to Undo (and Redo) Words with Facts: A Semio-enactivist Approach to Law, Space and Experience

**DOI:** 10.1007/s11196-022-09912-7

**Published:** 2022-06-11

**Authors:** Mario Ricca

**Affiliations:** University of Parma, Parma, Italy

**Keywords:** Law, Facts/values, Enactivism, Global semiotics, Categories, Legal theory

## Abstract

In this essay both the facts/values and facticity/normativity divides are considered from the perspective of global semiotics and with specific regard to the relationships between legal meaning and spatial scope of law’s experience. Through an examination of the inner and genetic projective significance of categorization, I will analyze the semantic dynamics of the descriptive parts comprising legal sentences in order to show the intermingling of factual and axiological/teleological categorizations in the unfolding of legal experience. Subsequently, I will emphasize the translational and enactive cognitive disposition underlying the construction of the second premise of the so-called judiciary syllogism and thereby the untenability of the idea that ‘law makes its facts.’ Hence, I will try to bring to the fore the cultural pre-assumptions encapsulated in the positivistic and therefore also formalistic or analytical approaches to legal experience and the loss of their inner consistency when legal experience confronts the phases and major changes of global semiotics. Finally, I will strive to relativize the opposition between the positivist and non-positivistic theories of law in view of an understanding of legal experience focused not only, or at least not primarily, on what ‘law is’ but also on ‘how’ it unwinds through, and *in spite of*, environmental and semantic transformations.

## Prologue

In this essay I will address the relationship between facticity and normativity in the attempt to proffer a processive and enactive approach to legal experience. In order to pursue this investigation plan, I will adopt a semiotic methodology combined with the enactive perspective first of all to analyze the significance of categorization. Subsequently I will try to give a relational portrayal of the processive intermingling between facts and words. This passage will be performed in order to bring to the surface the interpenetrative relation extant between ‘matter’ and ‘sign’ as well as the semiotic continuity dynamically interweaving these two domains and stoking the ‘emergence’ of meaning. Subsequently, I will propose a critical analysis of the semio-spatial implications of categorization with specific regard to the phrastic/descriptive parts of legal provisions and the translational activity involved in the elaboration of the second premise of the legal/judiciary syllogism. Hence, I will apply the results of the argumentative itinerary developed to the dynamic of legal systems through a contrastive examination of the various theoretical declinations of the positivist view of law. I will carry out this critical activity to test the operative consistency of positivist understandings and the operational ‘use’ of law when legal systems are to face the mobilization of the global semiotic landscape coextensive with the world of facts tacitly included in their rules. Thereafter I will strive to show how the positivist epistemological approach to law is necessarily embedded, as regards its operational consistency, with some socio-cultural and factual conditions that are vulnerable to global semiotics, and specifically those portions (presumptively) assumed and external to law. I will try to exploit these interlocutory outcomes in the endeavor to illustrate how a processive and enactive conception of legal processes and experience can reduce the blind spot of positivism, at least when the relational global semiotics surrounding both social and legal experience—as it were—*is set in motion*.

## Law and Global Semiotic Dynamics: The Interplay of Categorization, Values, Meaning and Space

As just mentioned, the orbital axis—as it were—of my enquiry is the relationship between categorization and spaces of experience. In this regard, I immediately observe that the determination of the boundaries of each category, taken in both its semantic and pro-active projections, is the result of evaluative choices. These maintain their stability provided that the boundary (semiotic and experiential) conditions do not change. In this respect, on the other hand, it is to be underscored that the same true-functional logic, or truth-value logic, cannot avoid relying on the presupposed existence of linguistic conventions or culturally founded rules for the determination of meanings and the subsumption of factual elements within categorical and propositional schemes. That said, at least in my eyes, the main question to address is: What happens when different universes of discourse come across one another and their *spaces* of experience and signification intermingle? And, therefore, do the related boundary conditions resting on top of categories fluctuate by virtue of a variation of the overall semiotic landscape through which experience unwinds? And still, what if different (cultural) ways of categorizing facts come into contact and interpenetrate because of environmental factors?

In the situations just outlined in an interrogative fashion, the determination of what is within each category and what is outside becomes an open question—although, at least in my view, it should also ordinarily remain open. This occurs because the renewed and unprecedented spaces of experience show a tendency to engender new relationships among elements endowed with signical and axiological relevance. This semiotic motion activates a relational dynamic among the same values underlying the previous determination of the extant categorical boundaries and, in many cases, rebounds on (the why of) their permanence. All of this rests on the presupposition that the world has no labels and that the categorical spectrums are the result of a carving out of semantic perimeters from the flux of the relational interplay between the mind–body units and the environment. Under the conditions just sketched out, the game played for the meaning and determination of the categorical structures necessarily begins again. This is also because rigidly applying past categorical schemes could generate a heterogenesis of ends and unbearable inconsistencies in the same use of categories [153].

Just to make clear the ground of my investigation, I would like to emphasize that, in my view, the axiological or teleological dimension is inner to the selection of the constituents of each category.^1^ Differently from many scholars,^2^ I think that the teleological-functional dimension is not heterologous if compared to the categorical structure (or the concept). I mean that it is impossible to assume that first the categories and concepts exist and then the mind identifies their function. In my view, this entails taking an untenable retrospective approach to the process leading to the formation of concepts/conceptions.^3^ Conversely, the axiological/teleological ‘moment’ is internal, in the sense that it determines the categorical structure by defining, respectively, on which elements to place the salience axis of each category’s semantic scope, what to consider central and peripheral, what to include and what to exclude from the categorical frame or relational diagrammatic figuration,^4^ and so on. From this perspective, when the boundary conditions change, the use of the previous categorical structures can run into both axio-telic dysfunction and logical inconsistencies. They can result, respectively, from analogical constraints that are determined by the commonality/continuity of properties between the elements previously included and/or excluded from each categorical frame. This is precisely the problem of inter-categoriality, which also arises within stable experiential and cultural contexts but becomes crucial when the spaces of experience—which are also semiotic landscapes hosting legal discourses—come into contact.^5^

Categories are semiotic entities, which, in turn, are composed of signs. Moreover, the significance of each sign depends on its relations with other signs and retrospectively results from them. It is not amenable to self-bounded compartmentalization. Such impossibility is part and parcel of the *exceedance* and/or *extopicity*^6^ intrinsic to each sign and, therefore, also to categories. From this point of view, the changing of experiential semiotic-relational networks and the selection of them according to values/purposes becomes an intrinsic problem for each circuit of signification. In these conditions, truth-value logic loses its cultural background. Somehow, space, categories and meanings—considered in their semiotic dimension—must undergo a reflexive and transactional re-adjustment process in order to not collapse in on themselves and their silent cultural semiogenetic prerequisites.

I think that the introductory observations presented so far about categorization (at large) also apply (and should be applied) to law and legal language. And this, more specifically, insofar as the categorical elements included in the phrastic part of the normative propositions always and inevitably show their genetic functionalization with respect to values, also because the connection with values is intrinsic to the genesis and the structure of deontic propositions. In saying this, I mean that the relationship between categorical boundaries and the axiological/teleological relevance of the elements included in them is explicit. In normative language ‘is’ always implies, at least indirectly, an ‘ought to be’ and hence an inner relation to values/ends. By contrast, the same cannot be said of purely descriptive/empirical language and categories, where the connection with values/ends is implicit and, in a sense, epistemically removed along the process leading to the construction of ‘objectivity.’ However, in both cases, axiotelic factors are still present in phrastic and/or empirical categories but can be unveiled only through a work of anamnestic semiotic *dis-composition*. A task which with regard to the ‘descriptive/constative’ language, especially with regard to the factual elements, is more difficult to be carried out. As just intimated, the reason for this increased difficulty is that the axiotelic components are ‘rendered’ tacit by the apparent objectivity of the empirical data. And this because their ‘empiricity or factuality’ results, in turn, from the embodiment of the process of categorization and its purposes within the categorical structures and even in the same sensorial perception of the factual ‘Given’.^7^ The relevance and the ‘thickness’ of the embodiment processes becomes distinctively clear when one considers that the space of experience of each individual is first and foremost a semiotic space. As such it is coextensive with the signical landscapes drawn by virtue of mnestic activity and its attitude to interactively presentify past and present, as well as multiple reciprocal ‘elsewheres.’ For any individual’s life environment is independent from geographical distances and can make semantically close what is physically remote, and vice versa. The production of sensorial perception dynamically epitomizes and rearticulates such semiotic texture so that what is perceived ‘here’ and ‘now’ forms a ubiquitarian space/time tapestry. This is, inter alia, the reason why the dynamics of global semiotics impinge on the relational interpretation of the founding values/ends of every legal system and, in this way, on the boundaries of the categories included in the phrastic parts of legal rules and their semantic frames (namely, with regard what is to be included or excluded from their structural boundaries).

My goal in this essay is to trigger a dialogue between different theoretical approaches—respectively, analytical, hermeneutical, pragmatist—with reference to (a) what I call the game of ‘meaning production’ or *poiesis*; and (b) the need to keep this game semiotically open and consider such openness as an intrinsic requirement of any legal system at least for reasons of both consistency and effectiveness and their reciprocal relations. I think that such a dialogue, if successfully carried out, could have considerable implications for the very way of conceiving the same analytical approach and topics such as legal certainty, the condition of logical-legal inference and, above all, the critical assessment of the construction of the *medium* term (second premise) of the judicial syllogism, especially with regard to intercultural relations. With regard to these topics, I am particularly interested in the consequences for legal theory stemming from the assumption of the relevance of global semiotics as a lens for a renewed analysis of the relationship between ‘facticity’ and ‘normativity’ in legal experience. In order to preliminarily sequence the main issues raised and involved through the adoption of this perspectival angle I would like to list a series a questions and interlocutory considerations designed, *inter alia*, to outline in some way the state of the art in this field.

Does the consideration of a global semiotics of contemporary socio-legal experience and its intercultural spaces impinge on the traditional positivist-inspired interplay between ‘facticity’ and ‘normativity’? And, more specifically, to what extent does this affection—if any—retroact on the semantics of law as well as the prerequisites for the legitimation of legal systems? As is well known, the traditional positivist approach to the relationship between facts and legal rules is grounded on the ‘quasi-mantric’ assumption that ‘law makes its own facts.’^8^ This interpretative axiom, in turn, stems from the conviction that deontic languages, including the legal one, constitutively determine and select what is relevant for their universes of discourse and their pragmatic projections. Needless to say, both these postulations rely upon the is/ought divide and the rejection of any kind of moral consequentialism.

And yet, how much do the above tenets of positivism—as well as analytical jurisprudence—rest on a socio- communicative pre-condition that takes for granted the ‘subsistence’ of a homogeneous cultural environment and a kind of semantic social contract? To what extent does the is/ought divide rely on the pre-existence of an assumed (almost mythical or axiomatically presupposed) objectification of what facts are—as such, separated and independent from values? Is this divide not rooted in the epistemology of Western secularization and the objective/subjective divide that stems from an absolute distinction between external forum and internal forum?^9^ Does this not result in a tacit ontologization of the products provided by an equally silent semantic social contract about those facts and the related categorizations?^10^

What happens, firstly from an epistemological point of view, if the cultural conditions underlying the ‘validity’ of the semantic social contract expire, or no longer apply to a given experiential and communicative environment?^11^ Insofar as these cultural boundary conditions underpinning the objective meaning of facts undergo a change, are the categorical assumptions concerning the factual dimension to be dismissed, or abandoned? And, consequentially, what about the role they play inside the phrastic/descriptive parts of legal rules? This last issue can become even thornier if we consider that the ‘facts’ legal language relates to are not only products of a self-referential linguistic function, as could be said of the terms ‘contract’, ‘inheritance’, ‘corporation’, ‘property’, etc. On the contrary, these categories are embedded—often implicitly—with innumerable other categorizations pertaining to or drawn from other ‘non-legal’ languages (including the so-called ‘natural language’: *whatever it may be*). To put it more bluntly, they cannot be defined as ‘purely legal ones.’ This means that when the extra-legal categories regarding the objective world of facts are to be re-semanticized (and, in a sense, ‘re—made’)^12^ because of the transformations of the experiential world and its semiotic relational landscape, this change will inevitably rebound on the legal discourse. At that point, won’t the extra-legal ‘facts’ (encapsulated in law’s discourse) and the cultural/axiological determinants of their alleged objectivity influence the legal values, their reciprocal relationships and, subsequently, the categorical frames of ‘legal facts’?

A global semiotics of contemporary social and legal experience is coextensive with the interaction among multiple ‘worlds of facts’ as well as different axiological ones. Consequentially, when molding what is relevant in the phenomenal world for its own ends against the foil of this global interplay, the legal language can no longer assume as extant a bounded experiential and cultural circuit coextensive with the socio-communicative stability ground—as it were—of a semantic social contract. Therefore, law cannot presume the existence of an axiologically neutral dimension of extra-legal facts, the same facts that it however includes. On the contrary, the subjectivity/value-laden origin of the multifarious ‘factualities’ at stake cannot remain encapsulated and, in a sense, dissimulated by a (culturally) taken-for-granted objectivity of ‘the World.’

The ‘Fact’, in tune with its etymological origin (factum, from Latin ‘facere’), unveils that its assumed objective existence is something ‘made’ (precisely: ‘factum’) by someone: in other words, it is not an original unauthored ‘datum’ but a proactive^13^ and enactive^14^ ‘result.’ This implies that anything that is assumed as a fact constitutes, in any case, the morphological emergence of an underlying relationship, or better, an ongoing relational process of which the categorical morphology is only one interlocutory stable state (relative, and thereby silently morphing). Representations of facts and their categorical schemas are only that which remains of human experience (in the form of cognitive/behavioral scripts, habits and schemata) filtered out of a semiotic dynamic stream after the subtraction of the subjective/proactive part of the phenomenal relationship.

The preceding remarks suggest that the semantic social contract by which the (cultural) objective world of facts is kept afloat does not engender a neutral picture of something extant ‘out there.’ Conversely, it produces a collection of categories consisting of enactive projections historically and proactively gushing out from the ongoing and unremitting relational interplay between the subject/organism and the environment surrounding its living action. Each representation and the morphological categorical features it includes, function, in turn, as instruments supporting the adaptive re-enaction of the organism/environment relationship. From this perspective, morphological ‘figurations’ and categories can be thought of as proactive epitomes that bridge past and future relational experiences in a transformative way. Their meaning and the coherence of the overall knowledge system is a consequence of the future transactions taking place on the edge of the renewing of experience. Objective worlds stemming from those transactions are, therefore, not only subjective representations but also constituents of existing worlds, a substantial portion of which, however, remains invisible, silent, implicit, or beyond the frontiers of representations. Exactly this semiotic exceedance^15^ requires that the interaction between different cultural worlds or the alteration of the environmental boundary conditions surrounding previous categorizations lead to a redefinition of the spaces of implicitness/invisibility behind semantic categories. This semiotic renewing necessarily entails, however, a translational effort between the involved ‘spaces of invisibility/implicitness’, which in turn brings a transformation of the categorical morphological features. Once again, it is to be emphasized that the semiotic landscapes underlying, and epitomized by, factual categories are composed of elements spread across time and geographical space. As a consequence, the objective meaning of what is perceived as present ‘here’ and ‘now’ turns out to be imbued with multiple time and spatial ‘elsewheres’ or ‘remoteness.’ This implies, moreover, that the more culturally different people and phenomena of various sorts (biological, geological, etc.) come into contact, the more such multiple otherness and their semiotic clouds interpenetrate, generating a constellation of thirdness from the ‘height’ of which past categorizations must be ‘re-semanticized’ and systematically made ‘consistent’ just to be kept consistent with their generative gist.

What, then, of values/ends when they are regarded against the above dynamic background? What is their role in the just outlined process of experiential and semantic transfiguration? In an environment coextensive with global semiotics, *manifold objective worlds* will interplay with each other. But, in such a situation, any choice about which properties and connotations to include in the individual and or social acts of categorization will have to measure itself against the exceeding axiological relevance of what remains excluded each time by the categorical boundaries drawn to qualify the factual situations and/or items. To put it more simply, any property or connotation excluded from a categorical perimeter could just as easily be subsumed under the same values/ends orienting the inclusion of something else within one or another category. This ambiguity is the phenomenal emergence of the inevitability of inter-categoriality and the related tendency of the semantic texture of language and cognitive schemas to continually give rise to metaphorical transformative and relational readjustments or amalgam among them.

The dynamics triggered by the above-described semiotic exceedance projects on the semantic of values consequences that extend also to legal systems. More specifically, from a legal and axiological point of view, it can convey the involvement of values/ends different from those considered by legal languages or authorities when defining the array of aspects of the phenomenal world that are relevant for a specific/local law or legal system. But this involvement of other values can easily turn into a rearticulation of the extant relationship inside the overall axiological texture and the respective meaning of their components. The ensuing interplay and interrelationship among values can end up redefining, in turn: (a) the semantic scope of each and (b), as in a kind of semantic roundabout, the way such values will affect the selection of properties and connotations to be assumed in the definition by legal discourse as well as other languages the semantic cartography of the factual objective world. Actually, the values on which the legitimacy of legal systems are culturally and politically grounded are ultimately ‘continuous’ and coextensive with those encapsulated in the ‘semantic social contract’ underpinning other languages, including the natural one.^16^

When intercultural as well as inter-natural inter-spaces of experience and signification take shape, they function as cradles (or, in Plato’s words: receptacles—see *Timaeus* and the idea of chóra) for a renewed threshold of the world(s) objectivity.^17^ What springs from this intermingling of semiotic spaces are renewed ‘factualities.’ The rise of this new arrays of ‘facts’ presupposes the disappearance/subtraction, but in a sense also the dissimulation, of their subjective/axiological components and the establishment of a new inter-subjective communicative stage of experience. What makes this possible is the upgrading of the semantic social contract, which involves also a reinstatement of the semantic constituents of the is/ought and values/facts divides. Such kinds of processes undergird even the logic of positivist and/or analytical jurisprudence and practice if only because a durable effectiveness of the semantic social contract is one of their crucial pre-conditions. The state of stability will only last, however, until the relational mobility of the semiotic webs reactivates again as a consequence of (a) the inherently transformative and enactive dynamics of the interplay among the environmental factors; or (b) the occurrence of abrupt upheavals. A kind of cyclic process of transformation^18^ underlying the phenomenal unfolding of legal experience thus looms, due to the inherent openness and transactional^19^ origin of categorizations as well as their constitutive attempt to grasp the future.

The question that arises, therefore, is whether the study of the just sketched out features/factors of legal experience should remain outside the province of legal theory as well as the practice of law, as the positivist, formalistic and analytical approaches seem to contend; or, instead, if the allegedly ‘descriptive’/scientific assumptions behind the positivist ‘concept of law’ are a mere consequence of a particular semio-ideological^20^ understanding of modern categorizations and its *affixing* onto the legal experience.

## Reversing Latour’s Reverse of Austin’s ‘Making Things with Words’: Undoing (Legal) Words Through Facts

Quite a few years ago, Bruno Latour wrote an essay entitled ‘The Berlin Key’ [100]. Paraphrasing Austin, but only to critically overturn his thought on performatives, he subtitled the essay: ‘How to Make Words with Things.’ Taking as a starting point the cumbersome use of the Berlin key, which requires the active collaboration of a circuit of subjects in order to perform its function as a key, Latour wanted to demonstrate how things could contribute to the making of meaning of words and the semiotic weft of social communication. More specifically, his goal was to show how the meaning of the ‘Berlin key’ was neither in the word nor in its morphological characteristics of its thinghood, but rather in the generative relationships between that ‘thing’ and the coordinated, and always to be rearranged, activities of the people involved in the gesture of ‘entering the door.’ In this way, he sought to emphasize how the functioning of the key varied according to the spaces and the times through which people carried out the roles that were orchestrated and pivoted around that object; and most importantly, the ways in which people renegotiated their roles by virtue of its morphological and interactive features.

The title of this essay is designed to suggest the possibility to reverse the arguments through which Latour overturned Austin, so as to approach in a processive way the relationship between normativity and facticity with specific regard to legal experience. My purpose is precisely to show ‘How words are undone by facts… to be redone by virtue of the semiotic interaction with them.’ That the semantic weft of words is continually unraveled and rewoven I believe to be a phenomenon that humans experience every day. And if it happens, fortunately, it is by virtue of the *fact* that the categories included in any sentence—whether they be deontic or descriptive—refer to semio/pragmatic and inherently relational contexts; moreover, they epitomize, both retrospectively and prospectively, the related contextual relationships. This approach induces the conception of categories as means designed to capture the future; to do so, they can only imply a coefficient of entropy, therefore a certain degree openness towards transformation and transaction according to the unfolding of experience and its semiotic implications.^21^ From this perspective, what Latour observes in the essay cited above appears to be very in tune with my argument. He noticed [100: p. 18] that, from one angle, it could be said that ‘technology is nothing more than discourse, totally expressible in other media.’ He refers, however, to the interpretation of technological devices as *intermediaries* between pre-given respects of social relationships that, as factual fragments of the experiential and communicative universe, exist per se and are only connected, while still preserving their own individual and ‘exclusive’ identity/meaning. I reverse this assertion by saying that law, if assumed in its allegedly pregiven self-referentiality, boils down to nothing more than an *intermediary* of a presumptively ossified factual landscape and experiential discourse. As such it would be totally expressible (and, in a sense, should be expressed) as a material assemblage isomorphic to its conceptual meaning and the behavioral schemes it configures as the content of its provisions. When it is understood in this way, law is exactly as things *inter-*mediated by actions are.

On the contrary, the ‘expression’ of legal experience cannot avoid being dynamic, rather than petrified in pre-boxed semantic and pragmatic compartments. And, as paradoxical as it may appear, that expression restarts each day also by undoing the meaning of legal words as they are involved and semiotically transfigured in the production of factual experience, and vice versa. In the same vein, as regards the *mediation* activity performed by the Berlin key, as well as other objects and artifacts, if that word [mediation] were to ‘fill out a little’ [100: p. 19], then everything would change.^22^ And this because the meaning of what is *mediated* would no longer be transported only by the material medium, but also co-construed by its active involvement in the production of the dynamic relationships orbiting it. Such ‘being involved’ indicates, in other words, that the portion of meaningful landscape somehow hinged upon and expressed by the medium is mutually modified, changed by, as well as blended and intermingled with, other signs and semio-experiential networks.^23^

Now, if we transpose Latour’s argument about the Berlin key, we can say that if facts fill out a little with semiotic significance, they concur to determine the meaning of legal words. Of course, facts are relevant ‘in order to designate’ the action of legal words, even if the latter are not only passive targets of the ‘mediating function’ of facts and their relational/signical bearings. On the contrary, between facts and legal words there will be a continuous process of semiotic co-pollination and inter-penetrative interplay. What is important to emphasize, in this respect, is that when facts change, the semantic scope of legal words also changes. On the other hand, the argument that such a change should not be too extended, so that the prescriptive ‘nature’ of legal enunciations—their deontic hallmark—is preserved misses the point entirely. This is because prescriptions can be obeyed and engender obligation only insofar as they are in tune with the values/ends of the legal system, the same one that legitimizes them. Hence, if facts are part and parcel, as *mediators*, of the *mean*-ing of legal words, they will also be constitutive of the meaning of values/ends lying at the apex of legal systems. But, in turn, these values/ends are inherently constitutive of the semantic scope of legal sentences, including their phrastic parts. If this is correct, then the semiotic relevance nestled in the mediating function of facts will co-constitute the scope of the phrastic categories included in legal utterances. This is because the variations in the ‘factual dimension’ and/or its semiotic reticular/relational composition should be taken as inherently immanent in, or continuous with, the semantic reach of legal words. In short, there is no a priori and *pure* dimension of significance of the legal system and its provisions independent from their embedding in the co-constitutive interplay between facts and the values/ends that constitutes the legitimacy of law. But the last assertion is to be considered both cognitively and axiologically valid only to the extent that values are taken as processive means in the making of facts, and vice versa. Which is to say that values and facts are to be taken as processive and interplaying ‘means’ of the ongoing unfolding of social experience conceived in holistic terms.

Law cannot be construed with the law and also social facts need law to become what they are. This is the reason why one needs the other, and vice versa.^24^ The ‘legal’ needs the social, the experiential, even because otherwise it would run the risk of becoming obsolete or end up completely detached from the world of experience from the outset. In this sense, we could say that law is, in turn, a *medium* between intention and experience, and knowledge mediates between law and facts/experience; and, still, experience mediates between law and the final result, which is the *socio-cultural concrete situation* forged by the application of law. Law and experience interpenetrate but all together mediate between intentions and final results (from an angle, by organizing and somehow controlling the reactions of the others who, in turn, make up the overall contexts). Intentions, as such, are nestled in both the Berlin key and the behavioral attitudes adopted by social actors involved in its use. As such they are invisible and yet, as signs, acquire materiality and explicitness through the dynamic process that takes empirical shape by virtue of their co-constitutive influence in steering social actors’ actions and relationships. From this point of view the meaning of both things and human activity borrows its ‘stuff’ from the realm of invisibility, which is a signical domain. There is no difference between the semiotic basin overshadowed by the morphological appearances/features of things and human intention lying behind individual or collective actions. Even the distinction between primary and secondary properties, if observed from the perspective being suggested, falls to the wayside or at least proves to be only a cultural heuristic device rather than an ontological distinction.^25^ In a sense, any object or fact is partially concealed and invisible, precisely like emotions, sentiments, ‘Moore’s good or yellow,’^26^ the internal perception of pain (which so concerned Wittgenstein)^27^ and values/ends. Understanding an object without the referral to the *mental* embodied in it, the plan of implications and actions that its concept epitomizes, is simply impossible. From this angle, there is no radical or ontological difference in looking after empirical objects or mental states insofar as both are clues of one another. On the other hand, any understanding of mental states, as moral judgments, involves in any case a context populated by objects, actions, namely external entities, which, however, are external only partially, as just observed. Internum and externum, psychological and material, mental and empirical are all signs, and owe their signification to something invisible: that is to say, the play of other signs that takes place in human (as well as animal) brains.

It is such continuity that makes it so that values and facts bi-directionally interpenetrate. Through the mediation of signical function the mental embodies the factual/empirical; in turn, the external/empirical incorporates the mental projections, also by virtue of the human actions that channel the mental toward the external and its transformations produced through the proactive and enactive projections of the mental.

Moore’s objections are undergirded by the peculiar and intuitive feature of moral judgments also because of their ‘invisibility.’ And yet, from the above perspective, they seem to find their linchpin in nothing but a mere prejudice: namely, that empirical judgments are construed only on the basis of the visible and not the invisible, that is, the signical landscape underlying visibility. It is exactly for this reason, I think, that Geach’s [61] linguistic comment works against [131] his assumptions. I refer to Geach’s observation that the moral or esthetical judgments, when strongly or intensively contextualized (I would add, *explicitly* strongly contextualized), seem to neutralize the is/ought, or facts/values, divide.^28^ In my view, the explicit contextualization makes so that the inter-relationships underlying both factual and moral/aesthetic judgments are made visible/explicit. In this way, it contributes to dialectically forge the meaning of both the factual and the moral/aesthetic/qualitive assertions. Those inter-relationships, in a sense reciprocally ‘co-graft’ facts and values, as well as the respective semiotic constituents, so that such a semiotic web engenders continuity between the semantics of facts and values. Any change or new choice in one of these domains will originate a shifting in the other, and vice versa: and all this goes on until a newly weighted combination is reached. The main difficulty—following [61]—to find the explicitly contextualized moral judgment with regard to human beings, depends on freedom and the open indeterminacy of the contexts that human beings are able to generate. But this is only a limit stemming from, and coinciding with, our ignorance; as such it is not rooted in ontological or cognitive differences, and even less is it due to some conceptual status.

All the above considerations apply also to law and, more precisely, its relationships and ‘trade’ with experience. As I try to illustrate below, Hart—just to give an example—when speaking of the ‘open texture’ of (legal) language, makes a huge mistake insofar as he thinks that that ‘openness’ is a marginal feature of law, which applies, as such, only to specific unrelated terms or expressions. Conversely, each word is open, each case can become a ‘hard case’ and be expulsed from the ‘semantic corral’ of *normality*. This is the reason why the relationship with facts and their signical as well as axiological ‘matter’ can *undo the word*s and their pre-packaged, stable signification.

Facts as well as the Berlin key are not only the constricted material boundaries that apparently define their empirical perimeter. On the contrary, they and their *mean*-ings include the semiotic network encompassing the possible relationships and threads that—whether retrospectively or prospectively—are engendered by and pass through them. In short, everything is a signical crossroads; signification lies beyond the contours of what is perceptible as being ‘here’ and ‘now’, so to speak, beyond its ‘visibility’ or, more generally, its immediate perceivability.

As signs facts, and the same space to which they are immanent, are not different from words in that they are placed and interplay through an interface of dynamic and relational continuity with one another. Consequentially, the semiotic ‘matter’ of facts constitutively and enactively interpenetrates with the ‘semantic substance’ of words. This implies that then the more the semiotic weft of facts is spread through and dynamically interwoven with *relational elsewheres*, the more the meaning of words is to be conceived in responsive terms to this ‘remoteness.’ Such responsivity includes the self-transformation needed to ensure that the continuity between words and facts, and their underlying semiotic webs, is reciprocally integrated on both sides, at least asymptotically. If we apply this view to legal experience, and namely to legal words and facts, it immediately becomes clear that the dynamic of global semiotics or the reciprocal interplay of manifold ‘elsewheres’ is inherent to the ‘signification’ of each ‘local’ and its apparently bounded experiential circuit. This conclusion sheds new light on the issue of the legitimacy of legal systems and its ties with the allegedly biunivocal correspondence between legal space and ‘physical’ territoriality, not to mention its self-bounded ethno-cultural scope. Insofar as the ‘elsewhere’ is factually/semiotically inner to the significance of every ‘local dimension’ and the correspondent activity of the cultural semanticization of its spatiality, the meaning of legal words cannot shirk the inner transformation stemming from the dynamics of global semiotic interpenetration.

In view of these interlocutory outcomes, resulting from a processive/enactive reading of categorization and its spatial relational bearings regarding legal experience and semantics,^29^ some crucial question arise about the validity preconditions of truth-value logic and its conventional prerequisite, especially as concerns their use within the formalistic and analytical approach to legal theory. The source of such questions is the axiomatic necessity that compels the true-functional logic which assumes the pre-existence of a well-established conventional semantic *setup*. An idea which, *mutated mutandis,* serves as a cornerstone also for the positivistic logic of legal validity, as such embraced by both formalistic and analytical approaches to legal experience as well as, to a certain degree, by the various strains of normative/ethical discursive and interpretive theories of law. Nonetheless, the ‘conventional’ hypothesis functions only as long as the cultural and thereby semiotic warp of facts can be taken as coextensive with a cultural and experiential domain hemmed in by almost granitic borders unrelated to any other ‘elsewhere.’ This constructive option, however, can be heuristically assumed as useful and plausible only so long as the dynamics of global semiotics is contingently unvarying. Which is to say—to use my own phrasing—that the ‘invisibility’ of the relational underlying landscape of things does not set in motion and reveal its ‘silent co-implication’ in what is assumed as ‘the visible’ and its surfacing features. In these cases, the inability of legal systems to maintain the preconditions of their own existence and validity becomes not only evident but also unbearable, if not socially and cognitively pathogenic. The point is, therefore, not so much to raise doubts about the absolute theoretical plausibility of positivistic ‘renderings’ of legal experience and legal systems as rather to determine whether these approaches are useful when global semiotics is in turmoil. In the same vein, the problem to confront, in such situations, is whether legal systems positivistically conceived and ‘operated’ are suited to control a space of experience, and therefore a *factual* one, that is overdetermined by external factors for its unfolding and meaning. Or if, instead, their being anchored to the semantic stiffness that is constructively germane to the hierarchical and past-based logic of positivism does not make them collapse in on themselves, thereby frustrating the processive and semantic openness of their own foundational values/ends. And, just to push the inquiry on a little further, it is also questionable whether both the positivistic and the ethical discursive approaches, especially with regard to the elaboration of the second premise of legal syllogisms, are suitable and cognitively powerful enough to manage the interpenetration between different cultural spaces/universes of discourses and the related intercultural and thereby *inter-*factual dynamics. In short, are the ‘positivistic legal systems’ or the ‘systems grounded on an ethical/normative self-reflexive rationality’ able to support an intercultural translation of reciprocal ‘elsewheres’ in the interpretation of their own categories and the achievement of their foundational values/ends? Is the binomial ‘truth-value logic/hierarchical rationality’ inherent in the theoretical and ideological imaginaries that is coextensive with positivism and normative/ethical rationality semiotically equipped to undergo the awakening of global semiotics and the fading of its previous (relative) stability? And still, are legal positivism and its underlying cognitive and cultural imagery in tune with our times and the global semiotics circling around what is mapped and carved out from the unbounded continuity of the spatial dimension known as ‘local’? Or, instead, is the semantic algidity of hierarchical logic inherent in the positivist and ethical/normative theories of legal experience and institutions an obsolescent smokescreen that conceals a tendency to eternalize the historical/cultural script coexstensive to the epoch of national states and the related spatial imageries? In the following I will try to reduce, at least, some of the theoretical and pragmatic blind spots collateral to the enduring predominance of positivism and the current fortune of the ethical rationalism in coping with the increasing mobilization and renewing of the global semiotic space. I will also attempt to show: (a) that tacit socio-cultural presuppositions of the various positivist approaches to law engender a communitarian/conventionalist as well as territorially compartimentalizing semanticization of space; (b) that the co-implication between corralled spaces of signification and legal meanings constitutes a cognitive hindrance to reaching an up to date attunement between the contemporary global communicative fluxes of people’s daily lives and the way both political and legal institutions operate. In this sense, I will strive to emphasize how and why the values/facts divide should be considered as an epistemological contributing cause of the inconsistencies extant at the core of the interplay between the global and the local circuits of legal, political, and anthropological experience. And this without leaving out how much the absence of a creative understanding and activation of the semiotic interpenetration between facts and values, first of all on a cognitive level, dooms the processes of functional differentiation^30^ to produce a *mere* hypertrophic and self-defeating increase of complexity and conflicts in the overall social and ecological planetary system. And besides, the divorce between values and experience, too often advocated in the name of a misleading and self-deceptive idea of freedom, along with the unawareness of and even the aversion to the inter-connectedness of everything and everyone, inevitably leads to irresoluteness and undecidability; which, in turn, is followed on average by the subsequent abdication of reason on behalf of a short-sighted and alienated competition for reified goals, the progressive outfall of which is an inchoate descent towards authoritarianism and its bellicose epiphenoma.^31^ But going back to the original question: are legal systems, by and large, able to maintain the pre-condition of their own validity and, in the end, existence when facing the mobilization of global semiotics and, therefore, the relational background of their attitude to impose obligations to constitute and protect individuals’ rights and prerogatives?

## The Misoneism of Positivism’ Logic *vs.* the Processivity of Categorizations

As I have already observed, legal systems (and especially the constitutional-democratic one) cannot rely exclusively on an axio-political social contract. They also need to presuppose a (pre-) extant semantic social contract which can serve as a ground upon which to construct negotiations of values and political ends. This duplicity of communicative domains does not imply that each of them is unrelated to the other. Social environment is construed through plans of actions that require the selection of the related means. But selecting means also entails a molding of *mean-ing-s*, which in turn will comprise the context surrounding the ends inherent in the plans of action and, thereby, the *mean-ing-s* of those ends. In a sense, ‘ends’ is a loop concept^32^ [33, 34, 37, 78]. This is because as long as they are ‘in view’, the ends function as means prompting action. As such, they processively include the sifting of means and their semanticization in accordance with their functionality towards the related ends; and yet, the interplay among the means cumulatively draws the semantic features of the ‘achieved ends.’ This semantic and, at the same time, pragmatic process, when applied to legal experience, does not stop with the birth of the legal systems nor with the moment of their pre-legitimation. On the contrary, the above semio-pragmatic activity should also unfold through the legislative and judicial production/interpretation of the foundational principles and values/ends, especially in democratic societies. The source of this continuous and reflexive re-semantization of principles and values/ends is freedom, to be intended as a cognitive-spatial pro-active attitude [92] and not only as a mere behavioral repertoire of legally pre-defined material conducts [75, 77: p. 13; 76: p. 162; 166: p. 124; 82: pos. 1717]. In this sense, freedom is synonymous with the path of renewal inherent in values/ends and categories taken as instruments to manage the relationships between human subjects and environmental phenomena. It is, therefore, the source of an unremitting process generating the thirdness resulting from the interplay between human cognitive and behavioral habits, on one side, and feedback of their pro-active projections, on the other. In this processive sequence, freedom performs an aesthetic and musing [143: p. 436; 22] function that imaginatively brings up and fosters the everting of the meaningful potentialities which are already inside the existing categorical patterns. This semantic unwinding is precisely the outcome of the selection from among the semiotic elements populating the experiential landscape and re-signified by means of their creative/pro-active recombination. In turn, such adaptive activity involves a semiotic dis-composition and re-composition of previous categorical schemes and habits to draw iconic or figural spatial frames within which human conduct and the related values/ends are situated.

Contrary to the just outlined dynamics of meaning, the legal paleo-positivist imagery conversely assumed that legal systems owe their semantic completeness and rational/meaningful consistency to a presumptive correspondence between the concepts they comprise and the cultural/ethical (substantive) constitution of the related social fabric. This is the tacit, and yet apodictic, safeguard clause through which, in their view, the legitimacy of legal systems comes full circle.^33^ It is not too dissimilar to what can be said of the formalistic and/or analytical approaches^34^ insofar as they enframe the dynamics of law within a hierarchical logic that is semantically immanent in the systemic set of legal provisions. In these theoretical outlooks, both foundational values and the semantics of the world implicitly nestled in their signification paradoxically epitomize in advance their own prospective implications.^35^ In other words, constitutional values/ends and the semiotic ideology of the ‘existing,’ play the role of semantic and praxiological pertinence constraints, and therefore validity, for all the rules stemming from their power of legitimation/authorization. For example, both Kelsen’s *Grundnorm* and Hart’s *Rule of recognition*, despite their self-representation as general presuppositions and yardsticks for the validity of rules, wind up working as retrospective instruments of legitimation that merely justify the features that legal system dynamics will have already assumed. Despite their divergences, as well as the openness they both recognize in the dynamics of law, this occurs because of the ties in their approaches interlacing—as observed above—values and meanings, the axiological/teleological social contract and the semantic one. This is true for two different, even if related, reasons:

- Quite apart from the debate about a cognitivist or a non-cognitivist approach to values, positivists displace the issue of their significance/existence to a stage predating the configuration of legal systems’ legitimacy. The objectivity of these values/ends is presupposed, as is the knowledge of their significance. Otherwise, they could not be used as a measure to assess the validity of legal rules and, consequentially, the lawfulness of legal subjects’ behavior. Nonetheless both the objectivity of values/ends and the meaning of the related principled/normative expressions are to be intended as an epitome of the instrumental processes (law-making, interpretation, application, enforcement, etc.) leading to the identification of their meaning; these processes, in turn, include the overall contexts of means that, through their ongoing experiential interplay, shape the final signification of each value/end. However, if the meaning of values/ends is taken as an objective and logical yardstick to assess the validity of each rule composing the legal system, then the process of their signification should be considered as something that has taken place in advance. In other words, the logical hierarchy used implicitly involves a chronological one as well. Therefore it is either one or the other: the legal system—as noted above—is nothing but a corpse and its dynamics only the social playing out of an already written script; or, alternatively, the judgement about the validity of legal rules consists in a surreptitiously retrospective and looping device, which, *in fact*, remolds the meaning of the same presuppositions (*Grundnorm, Rule of recognition, Constitutional principles, *etc*.)* that should function as standards to assess the validity of legal rules.
- The meaning of the terms that legal rules include, insofar as they partake in a deontic system relying on foundational values/ends, is isomorphic to categorical spectrums in tune with those axiological/teleological standards. This connection is clearly elucidated by the decision implying a judicial review of legislation that declares the constitutional illegitimacy of a legal rule because, for example, it does not include some subjects or situations in its provision; or vice versa. In these cases, judges enlarge the categorical scope of the words making up the rule under examination by treating the semantic projections of those words as means functional to the accomplishment of constitutional values/ends and the related principles. Consequentially, if the meaning of those values/ends is to be assumed as objectively pre-defined (see above point *a*), their objectivity will also imply the predefinition of the semantic fabric of social language: that is, the world as it is to be signified and ultimately ‘is.’ But this objective world is precisely the inherent hidden and dark side of law’s exteriority: that is, its distinction from the subjective domain, which is also the ‘realm’ of freedom.

Positivism, formalism and analytical jurisprudence do not exclude changes within legal systems from the scope of their investigations. The production of rules and adjudications (in accordance with the different schemes related to the separation of powers in civil law and in common law) are changes in themselves. Such changes, however, must necessarily take place within the axiological and semantic frames coextensive with the precondition of the overall legal system’s validity. Furthermore, not even the interpretation of procedural rules concerning the production of law is immune from value-laden judgments involved, for example, in determining the scope of their competence. This is the reason for which the above theoretical approach on average copes with the problem of semantic indeterminacy of both values/ends and the empirical categorical schemes included in the phrastic parts of rules through a kind of *existentialist* institutional strategy [73, 90, 91]: a more recent example can be found in [172, 173]. Legal rules authorizing the production of other rules or legal judgements allow the institutional subject to determine the ‘real’ significance of values/ends and categories, namely their semantic spectrums.^36^ The solution proffered to the problem of indeterminacy of what is theoretically assumed to be objective and shared nosedives in nothing but a tautological effectiveness. A very interesting example of this existentialist configuration of the extant legal experience is traceable, for example, in [115: p. 129], precisely when he says that ‘the existence of laws depends upon their being established through the decisions of human beings in society.’ If this sentence escapes truism, it is only because of its ‘objectifying value’, or better its being oriented to retrospectively prop up the objectivity of the legal systems’ prerequisite of validity and existence. Needless to say, the ghost of Austin [7] floats behind this argumentative strategy. What is implicitly cocooned in its adoption is the Austinian conviction that legal rules and their meaning can be understood immediately, at least on average, and this regardless of any axiological and/or teleological assessment of their contents, viz. their semantic extent. Such a tenet, however, dissimulates, or more or less intentionally overlooks, that meanings and categories epitomize the means/ends process inherent in the adaptive function of any human cognitive and pragmatic activity. But—as already observed—taking concepts, meanings, categories as evidence bereft of any past is only a deliberate act of ungrateful ignorance about the experiential process preceding and producing their emersion [33, 36, 40, 140, 143: §3; 174]. From this perspective, the attempt to overcome the interweaving between cognition and evaluation carried out, at least in the legal domain, through the methodological distinction between law’s descriptive knowledge and law’s production should be deemed only as a deceptive sleight of hand. This is for two reasons, at least:

- Understanding the meaning of any word, whether it has axiological or empirical content, cannot avoid the implicit involvement of the knowledge of a contextual framework, which is in turn nothing but a collection of operational symbolic and material means (or signs) leading to the experiential and mental stage corresponding to the emersion of signification. And yet, the implicit context differs from one individual to another and, moreover, sometimes its implicitness needs a critical externalization in accordance with the variation of the boundary conditions of each act of interpretation and implementation of the word’s semantic implications (and, in the case of legal statements, their application). But sieving from the experiential flux the elements to be considered in fleshing out the context of signification is an undetermined operation, the result of which will unavoidably come from an axiological/teleological disposition (whether put forward consciously or unconsciously). Hence, there is no room for any absolute axiological neutrality in either the understanding of the values/ends inherent in the legal system and its rules or the meaning of phrastic parts of normative statements including or presupposing empirical categories. Values and meanings have a semiotic nub, which as such cannot be considered outside their intertwining with other signs. This is the reason for which any radical axiological relativism is as assertion of semiotic solipsism; in the same vein, semantic absoluteness equals tyrannical seclusion from any relational semiotic dynamics. However, where there is no semiotic relationship, there is no cognition and possibility of signification. Meaning and understanding depend on this relationship, but which signs will be conveyed in the ongoing experience is unpredictable within a given event/situation. Any genuine grasping of meaning cannot avoid creatively coming to grips with the novelty sprouting out from experience. As paradoxical as it may seem, there is no authentic certainty (not even a legal one) outside a weighed transformative *going through* the relational substrate of novelty, in many cases embodied by and stemming from an individual expression of freedom, originality, or self-differentiation.^37^ The image of a descriptive interpretive activity as being neutral involves, therefore, a kind of axiological solipsism and, simultaneously, a cognitive empirical absolutism or tyranny. Despite appearances, what is at stake has nothing to do with the subjective psychological/ideological attitude of an interpreter before an objective reality recognizable as such in all its factual features and placed ‘out there’ with respect to its cognitive activities. [34, 36, 40, 79, 124, 179]. Quite the opposite, the above imagined neutrality is nothing but an imperative constraint limiting the cognitive possibilities of that interpreter and coinciding with the acceptance of apodictic postulations about the meaning of values/ends and empirical categories established by authorities. In the end, a complete neutrality, based on the self-evident meaning of ‘exteriority,’ would coincide with the abdication of any cognitive and critical commitment to the knowledge of law. As I will show below, insofar as we assume a polarization between exteriority of law and the *forum internum*, namely the exclusion of the aesthetic relationship between freedom and cognition [25, 80, 92, 142, 183], we deny the very possibility of ‘knowing.’ On the other side, passing off the danger of partisanship in judgments as an immediate consequence of any role recognized to the *forum internum* and, thereby, the relational connection between ends and imagined means, value and represented meanings, would be tantamount to spelling out the existence of legal phenomena (and, ultimately, the overall society) without explaining anything.^38^
- The distinction between a descriptive knowledge of law and the production of law is, moreover, a dirty game insofar as it conceals the commitment to know rules that pertain even to those who produce rules and/or apply them. In principle, at least, when lawmakers enact legislative acts they must ‘objectively’ know the content of the values/ends that should serve as a compass for their activity and, at the same time, as a yardstick to assess the validity of their legislative statements. This cognitive element is ineliminable from the perception of the binding signification of law. For nothing could be fathomed as deontic or obligatory outside the possibility to not recognize it as such. Freedom is constitutively intrinsic to any chance to conceive an obligation (at least, from an internal point of view).^39^ This means that the generative passage from freedom to obligation is to be necessarily mediated by a cognitive moment that makes room for both the performance of a free choice and the exclusion of a deterministic necessity, which would annihilate freedom and, thereby, obligation in one fell swoop.^40^ Such remarks apply to citizens, judges and lawgivers alike. Diversely legislators’ activity would be completely irrational and the relatedness of legal provisions to their own prerequisites of validity would only be a retrospectively orchestrated semantic fiction. On the other hand, even assuming that legal theorists could elaborate a merely descriptive knowledge of law, this function would in any case be socially and culturally continuous to the descriptive knowledge that we have to postulate as prior—in abstract—to the activity of lawmakers. Analogous remarks, moreover, apply to judges and the application of legal rules. And besides, if it were cognitively and communicatively secluded from the activity of lawmakers, judges, legal practitioners and common people when interpreting law, the question ‘Who needs legal theory?’ as coarse as it may sound, would appear as unavoidable as unsurmountable. However, experience shows that this is not at all the case.

Some authors have become deeply aware of the above theoretical and pragmatic troubles. To do justice to MacCormick, his latest remarks about the value-laden nature of legal knowledge, far from being a mere sign of inconsistency in his theorical production, reveals instead his insightful sensitivity to social experience [112–114]. The outcome of this awareness, however, does not change the semantic horizon of the problems at stake. MacCormick’s solution shows a constructivist bent that draws near to the theory of moral/normative rationality and argumentation developed, even if with different nuances, by scholars from various theoretical areas such as [2, 46, 48, 50, 57, 69, 150, 151] and many others—just to mention a few in a truly enormous literature. Nonetheless, all these approaches rely upon some kind of axiological or semantic socio/empiric objectivity and exploit it to legitimize the extant political-institutions or legal systems, at least if considered in their abstract correspondence to the theoretical figure of democratic constitutionalism. Despite their openness to discussion and inclusive dialogue, these theoretical approaches do not unmoor from the idea that any justification molded in legal terms and supporting claims of individuals or groups must dovetail into objective or previously determined and socially cemented axiological standards. Moreover, these theories, in a sense, seem to minimize the possibility that carving out ‘the world of facts’ from the experiential and social domain can be a ground of dispute that blurs with the moral/normative one. They tend to solve the problem by means of self-exempting tactics which entrust to institutional power the solution of these semantic/axiological disputes [73, 91]. Others, as noted above, rely instead upon the alleged resolutive power of rational/moral argumentation/justification [2, 46, 48, 57, 69]: to mention only a few voices of the contemporary debate. They, however, do not develop theories and methodologies that are powerful enough for the translation of cultural difference in the pre-established semantics of legal language, nor in the semantic standards pertaining to the other languages [125], including the common/natural one, that law presupposes or is imbued with in the phrastic parts of its provisions. On the other hand, these political and legal theorists show a pervasive and constant inclination to emphasize the normativity of their theoretical presuppositions which would make little sense if they were not concerned with the need to keep empirical cognition apart from evaluation, facts from values/ends.^41^

A common feature of both the aforementioned approaches is the conception of law as a system of directives that legitimate themselves by means of rational justification provided by the interpreters in planning their conducts and intersubjective relationships. Although the non-positivist declinations of this idea are open to the deliberative and pluralistic participation of the legal subject to the interpretation of law, the balance they envisage between the different and alternative viewpoints rests on the assessment of their respective coherence with standards of moral rationality assumed as objective and universal. The postulated divide between values and facts, factual judgments and axiological statements, ends up, however, immunizing the meaning of these moral standards against any genuine dialectical interpenetration with the experiential dimension. The objectivity and universality of those axiological standards becomes, once again, perfectly symmetrical to the allegedly neutral objectivity of facts and their meaning. In this way, values and ends will not be assumed as means to continually and transactionally regenerate the meaning of human relationships with the world and Otherness, so as to give rise to the emergence of an inclusive thirdness. Rather, as it happens, the prepackaged and taken for granted meaning of facts will be exploited as the foil against which it is possible to orchestrate moral justifications that are always oriented toward the past and functionalized to its reiteration by means of legal decisions.^42^

Unfortunately, the pre-asserted independence of values/ends from the empirical facts can allow interpretation to be unmoored from a horizontal and mutual interplay with factual experience [92, 157]. At that point the self-referentiality of each legal system [67] and its discursive divorce from any Otherness (cognitive, linguistic, material, etc.) can be justified on account of the normatively postulated objectivity/universality of its own moral/legal standards. The meaning of the standards of validity and the values/ends they enshrine, by and by, will wind up conflating with the existing rules, legally qualified social facts and the judicial decisions made about them. Fatally, this inevitable conflation will lead to the passing off—sometimes even unawares—of the previous interpretations/implementations of the foundational values/ends as the prototypical ones, with the effect of caging the future in the past and secluding freedom and any genuine (and thereby also cognitive) difference from the possibility to ‘enter’ the legal world. Holistic coherence and critical reasoning shall measure against each other on the field of objectivity/neutrality and then, the objective meaning of facts, which silently encapsulate dominant cultural values, will decide the fate of the game. The assumed objectivity of values and the objectivity of facts, almost invisibly, will support and justify one another in subjectivizing and expelling from the spectrum of universality/neutrality freedom’s attempts to re-categorize experience as well as the reasoning of Others.^43^ Any decision can find its rational legitimation in something that is logically and chronologically antecedent. The universality of moral reason and the eternal present of law (it is not a coincidence that legal sentences always use the present tense) will seem to come together. Through and despite changes nevertheless hinged on a top-down hierarchical logic of legitimation and justification, law can retrospectively, and then conceptually, make itself equal in the mirror of the objectivity and continuity of its foundational values/ends.^44^ Whatever happens, the meaning and validity of law *will* lie, however, in its premises.^45^

The tautological immanence of law’s meaning is not at all alien to the positivist shore of legal theory. Just like Austin, Kelsen and Hart—the last of whom I will return to—the contemporary legal positivists and the vast majority of legal professionals and practitioners assume that the meaning of a rule can (and must) be grasped regardless of any sharing of its content. In their view, (for example, [172, 173] and his rather authoritative ‘planning theory of law’), the issue concerning the semantic spectrum of the terms included or presupposed by legal rules is out of the legal domain; or, alternatively, it is to be faced by resorting to social consciousness, habits, implicit common culture or, at least, social debate and convergence on the procedurally-achieved semantic preconditions of rules’ validity. In other words, whenever a possible instability of a cognitive empirical element looms in the field of legal disputes, the solution is always deferred to something that (necessarily) pre-dates law and the inner dynamic of the legal systems’ validity. On the other hand, should they choose a different way, the exteriority of law and its inherent epistemological-political project would lose their foothold and inevitably the whole positivist version of the modern legal edifice would be bound to collapse.

Outside the strict area of legal positivism, the pervasive and constant trend of emphasizing the normativity of the theoretical presuppositions which political philosophers and other social researchers put forward would make little sense if they were not concerned, almost obsessively, with the need to keep cognition apart from evaluation, facts from values/ends. The paradox underlying all these approaches is, however, that they somehow are not able (or do not want to) disengage from the grip of the is/ought and facts/values divide, and yet spend their noetic life trying to rationally account for something—namely, law or political theory—which stems from what they implicitly postulate is just a hairsbreadth above the abysm of irrationality.

A symptomatic example of this attitude can be retrieved, paradoxically, also among positivists, and specifically in Hart’s ‘internal point of view’, assumed by the author as a pivotal feature of law as legal experience [73]. As is well-known, the author focuses on this feature of law against a foil consisting of two of Austin’s [7] tenets: (a) that law’s function is to provide for sanctions to induce people to obey in order to avoid them; (b) that the validity and, ultimately, the existence of a legal system coincides with the existence of the social habit to obey law. Hart argues that the attempt to hinge law’s existence and validity on sanctions and habits has no explanatory power. Although habits are doubtless a constitutive element of normativity, if not its social source from a genealogical point of view [73, 144, 145],^46^ along with sanctions, they give only an external reason for which people obey the law. Behavioral continuity inherent in habits and fear of sanctions do not say anything about why people think that they must obey the law. According to Hart, people abide by law because they accept legal rules as binding standards of conduct, that is to say that people internalize the rules by following them as a constitutive element of a social system governed by law. Nonetheless, as emphasized above, Hart excludes that the acceptance of legal rules also means that people share their content or recognize them as means to achieve their individual or collective ends. Hart underscores that individuals can be moved by the most varied motivations in their compliance with legal rules, but they are not what matters in order to understand what law is and how it is understood by legal subjects. In short, people obey the law and its rules simply because they think that those rules are binding. And insofar as something is legal because people think that it is such, that will be legal, ultimately, because it is (considered) legal. This is tantamount to saying that since people believe they live in a society governed by law, which is the condition of existence of the legal dimension itself (Lexitania),^47^ they cannot avoid accepting rules and obligations enacted by the legitimate institutions, as a way of creating consistency with their beliefs [171: p. 1167].

That said, one could wonder if there is some room for the quest for rules’ meaning and, consequentially, the reasons people think they are binding. What about when individuals dispute the meaning of some rule? And the problematic cases in which the categorization of peoples’ behaviors in legal terms—that is the construction of the second premise of the judiciary syllogism—is dubious? Why should judges or the same people choose one categorization over another? Or argue for the meaning of a certain fact, or rule, rather than another one? Hart has no answer to proffer from a semantic point of view barring the usual misoneist resort to a semantic past that somehow plays the role of a safety valve for the case of dispute on meanings concerning both the axiological and the factual categories comprising legal rules.^48^ The average clarity of the terms used within the legal rules seems to be considered by Hart as an implicit feature of any existing legal system. Incidentally, it is to be stressed that from this point of view he was much less open than Kelsen, who considered a relative semantic stability as a precondition only of the legal system considered in formalistic dynamic terms and, consequentially, identified a limit to the explanatory reach of legal formalism with a high degree of semantic and axiological stiffness. According to Hart, semantic clarity and the ‘semantic why’ that explains how rules are ordinarily identified and followed is assured by the functional links between means and ends already achieved in the stage of Pre-normitania and Normitania.^49^ When conflicts on the meaning of rules arise, it will be the legal system and its procedural rules to tackle these disputes so as to determine the ‘right meaning.’ In all the *hard cases* in which this result will be logically impossible, judges will face moral problems that can be solved only by resorting to the social environment and its extant cultural resources. If such a case happens, however, it does not imply that the positivistic (understanding of) law collapses in on itself, and this just because it will have always been the legal system to have determined the constraints within which it is legitimate to manage any semantic undecidability. This means that law itself authorizes the legal interpreter to draw in social cultural resources to establish the meaning of facts and identify the rules to be applied.^50^

By making use of the ‘internal point of view’ argumentative device, Hart does not exclude that the ‘issue of meaning’ is relevant for legal practice; rather he tries to distinguish the formal binding signification of law from its functional and meaningful features. In his view, the dialectics between means and ends inherent in the production of meaning is to be taken as a ‘solved problem’, definitively embodied by the terms included in legal rules and the procedural guidelines that law provides to face it. In so doing, however, Hart fails in his attempt to overcome the dilution of ‘legality’ in social ‘effectiveness’ immanent in Austin’s focusing on habits as a precondition of both legal existence and validity. The intention to obey law called into play through the ‘internal point of view’ stands for a de-semanticized intentionality. It could be captured in the phrase, ‘*oboedio quia oboedio*.’ But this kind of obedience seems to describe the behavior of a group of hypnotized individuals or, alternatively, pre-configured automata, rather than human beings. Their understanding of the rule of conduct, namely what they are supposed to do, is syntactic rather than semantic; or better, their semantics boils down to a syntactic sequence of formal instructions completely detached from any contextual, relational and teleological cognition and awareness.^51^ The world supporting this ‘unfleshed’ understanding is taken for granted, already defined outside the realm of legal experience. The life of law and that which people carry out through it is only an unrelenting repetition of behavioral schemas the meaning of which evokes, again, a semantic corpse. Therefore, if we conceive the possibility that the legal system hosts debates about validity and meaning—as Hart himself obviously does envisage—the alternative implication is: a) that the outcomes of these debate are only a fiction ordered to merely restate what already has been determined in its meaning and experiential matter; or b) that all the construction proposed by Hart’s and most of the positivist, formalistic and analytical approaches to legal experience are utterly false and nothing but a rhetorical orchestration designed to retrospectively justify unpredictable and irrational changes, maybe resulting from mere exercises of power. One could say, on balance, that Austin’s dilution of legality in habits, including that of seeking to avoid sanctions, at least seems more consistent and *existentially* sincere. On the other hand, as I will try to show below, Hart’s idea of habit, insofar as it reduces it to an unaware attitude opposed to intentional ones, is rather moot, if only because it corresponds better to habitude. Moreover, stiffness and complete unawareness do not actually fit into the philosophical and psychological category of habit [30, 37, 177, 186].

What sharply connotes the theoretical approaches considered so far is, in my view, a tendency to ingrain law’s existence and validity in the past and, simultaneously, to lose sight of the future. This is an idiomatic and somehow paradoxical facet of the positivist gaze, at least if we consider that its standpoint coincides with axiological/moral neutrality/relativism and many of its theoretical projections aim at explaining the dynamic and diachronic unfurling of legal experience. In this regard, the main problem with positivists lies—I think—in their difficulties to include in their theoretical constructions the understanding that the legal system is unable, by itself, to maintain the preconditions of its existence and validity to the same extent that it is unable to determine them. This refusal prompts them to fetishize almost mystically the pre-legal dimension, which becomes, in a sense, the dumpster where they can discharge everything that is unpredictable, indeterminable, mutable and so on. This is a sort of out/out disposition by virtue of which everything that does not fit in their neatly formalistic and immanent ideal representation of legal experience is transformed into a pre-requisite of law’s existence: hence its analysis is outside the concern and competency of legal scholars. In so doing, however, positivists transform the understanding of law into an autoptic survey focused on something that can no longer transform.^52^

Law is a human artifact. Its meaning cannot be detached from the function it embodies and the teleological attitude that urges human beings to mold it.^53^ Understanding law involves the task of grasping the conditions whereby it can enfold the ends it embodies and reproduce itself as a social phenomenon. From this perspective, the inability of legal systems to maintain the condition of their existence and validity is to be considered not so much a limit to their rational and logical consistency (in this sense just the opposite of Hart’s thesis of the ‘open texture’) as rather a feature intrinsic in any cognitive and planned human activity. While remaining within the framework of Western modern thought, we can find the grounding for this consideration in Kant’s answer to the question: ‘Are a priori synthetic judgments possible?’ As Peirce [142: §2.690] insightfully observed in his reasoning on the relationship between knowledge and probability, the same question shook up the philosophers of that time. Kant’s answer, according to Peirce, was—more or less—that a priori synthetic judgments are possible insofar as everything which is taken as universally true is implied by the conditions of experience. Propositions such as ‘all bodies are subject to gravity’ or ‘all human beings are (at least potentially) able to speak’ cannot be inferred from experience. They assert something new and, in a sense, have a ‘positive’ and not only a ‘recapitulating’ scope. The blind spot intrinsic in the human ability to produce such propositions is that the conditions of experience cannot be taken for granted once and for all. We can only produce predictive hypotheses and revise them in light of experience but only by means of other further similar provisional statements, and so on. On the other hand, individuals’ supervening experience is always driven and molded by their own hypotheses as well as cognitive dispositions, which involve, in turn, cumulative transformative/transactive adjustments between human mind/body units and environmental factors. This means that human mind/body units’ reactions to environmental stimuli are never neutral but instead oriented by inner values, patterns and habits that are innate, developed and learned through experience and cultural communication. Therefore mind/body actions are dynamically and proactively oriented so that, insofar as possible, their enfolding implies a selective picking up and maneuvering of environmental elements serving as means to carry out experience. The experiential conditions that Kant pointed out as prerequisites for the very possibility of a priori synthetic judgments include thus also the axiological/teleological dispositions of human mind/body units. What follows from this feature of human predictive judgments and cognitive habits does not turn into an absence of objectivity or the legitimation of the tendency to assign inherent ends in cosmic reality or empirical phenomena—namely the enchanted pre-modern world [60, 185]. The more limited implication stemming from the foregoing observations coincides ‘simply’ with the inner teleological and ‘selective’ gist of cognition in both its symbolic and pragmatic respects. Through their adaptive cognitive means, human beings interplay with the environment by continually re-orienting their symbolic and practical activities and, gradually as well as self-reflexively, even the scope and the meaning of their values/ends. The domain of the ongoing and transforming relationships between mind/body units and other environmental factors (including other human subjects) is where meaning takes shape and place. Insofar as humans are unable to completely control or maintain the pre-conditions of the relational dimension in which their judgements and the related meanings emerge, human cognition can only reach a certain degree of probability, but never certainty or absolute truth. This is somehow widely accepted, even if mainly in a merely cosmetic way.

What is far less widespread is the idea that if we consider words and the categories they encapsulate as operating instructions for carrying out human activity (whether pragmatic or imaginative/symbolic), we can assume, then, that each word and category (as well as the syncategorematic terms: and, with, etc.) enshrines an a priori (implicit or explicit) synthetic judgment. Their scope is hypothetical, dynamically pro-active and connoted by probability, which suggests that their meaning and their revisability come together. If we exclude the teleological dimension from the understanding of words and categories, as well as the phenomenal knowledge they support, then we will inevitably fail to grasp the behaviors that living organisms and the whole material world entail in their action. Human ideas, plans, representations, categorizations and so on, are completely embedded in individuals’ activities and thereby in the relational and teleological experiential universe in which those activities are performed and which they contribute to producing.

We can consider the word ‘chair’ and the related category. Both of them concur to signify any possible ‘chair’ as an object but, even more so because it is an artifact, our attempts to catch its meaning would founder if we did not consider the dynamic, relational, teleological and material context which it currently as well as potentially embodies: that is, what could be called the experiential and semiotic cloud inherent in the chair and its meaning. The human mind and human activity are intermingled with the existence of a chair.^54^ A judgment irrespective of this co-productive and efficient contribution would miss the possibility to capture many of the meaningful implications of the ‘chair.’ On the contrary, we can say that we should try to grasp all those implications, both pragmatic and symbolic, that ‘our chair’ and its symbolic transductions epitomize and could portend. Our communicative habits ordinarily tend to silence and make implicit these semiotic implicational clouds underlying our representations and categorization. And yet, these relational, invisible implications are part and parcel of our understanding of the object either as an individual or as a member of a community that tacitly takes them for granted and expects from Others a skillful awareness in line with them. These silent and pro-active elements of representations and categorizations are as essential to our understanding of an object as the material and empirically evident ones. Their role comes to the fore as soon as people from different cultures happen to share the experience of some object the meaning of which is presumptively coincident with its material and sensorial features—metonymically speaking, with its ‘visible occurrence.’ In such cases, many pragmatic and discursive dissonances and discrepancies can take place and (often) generate conflicts unawares. Their source is ignorance of Other’s teleological horizon and its influence in picking out the features of the object at stake which are considered salient for its categorization and the related proactive projections, as well as the behavioral attitudes orbiting around it.

To efficaciously tackle such conflicts, what is to be realized is that there is no object *out there* and, on the subjective side, not even individual representations of it. Consider, rather, the production process of a ‘chair’, the construction plan and its inherent end in view. They determine the means which will play the role of properties and connotations of the realized objects, holistically considered. The meaning of the final object, as well as of human words and categories, will entail in itself all the semiotic clouds and implications it processively includes both retrospectively and prospectively. To put it briefly, the ends and means epitomized in the meaning of ‘chair’ create what the chair ‘is,’ the context of actions it engenders, and the related proactive spaces of experience, and not only how the ‘chair’ appears from a subjective perspective. All these elements, in turn, define a categorical checklist that goes beyond the merely apparent morphological properties, radially including all the possible semiotic implications, material and symbolic, retrospective and prospective. What is important to underscore is that the components of this enlarged categorical spectrum are not exclusive of one single category. On the contrary, their occurrence is distributed among multiple categories along a trans-categorical *continuum*, which conveys phenomena of inter-categoriality, metaphorization and trans-categorical blending and/or migration of individual items previously placed within some categorical boundaries rather than others [155, 159]. Nonetheless, even if one looks at a ‘chair’ as an external object and does not presently consider its being an ‘artifact’, namely a kind of mind-thing hybrid, her/his ‘intellectual apprehension’ of it will in any case be inter-active and interpenetrative. This means that any gaze on the world is ‘specious’ (echoing William James), driven by ends/values, dynamically relational and accompanied by the proactive processing of semiotic clouds engendering imaginative and potentially pragmatic experiential contextualizations. From this point of view, what has been observed so far with respect to artifacts also applies to the knowledge and even the perception of the entities and phenomena populating the *natural* world. Even if they are not productively integrated in the process of their production as artifacts, teleological elements are always present in human understandings of so-called ‘natural objects.’ The categorization of individual items as well as the previous creation process of categories is based on an activity of selection concerning what is to be considered salient within our cognitive relational experience with it; the judgment of salience depends on human values/ends. What a ‘stone’ is depends on our gaze on it, and the functional projections inherent in this gaze cannot be detached from the ontological ones. This is precisely because the ends/values steer the selection of the features to be considered salient in order to give shape to the categories or identify what categorical spectrum applies to the object—which corresponds to the determination of the second premise of syllogisms considered from a dynamic standpoint (in Peirce’s terms, the abductive phase of judgment).^55^

That said, it is to be emphasized that despite their ‘invisibility’, the semiotic clouds involved in categorizations would be erroneously intended were they to be labeled either as ‘subjective traits’ of the judgment of an object or a phenomenon, or as ‘internal connotations’ of an external factual occurrence. Those semiotic clouds, including ends/ends values and the related context of means, forge and are integrated in the ‘molar’ meaning of the entities populating the so-called external world. What makes them ‘invisible’ or morphologically implicit is only the result of an epistemic ellipse that takes place when the experiential relationship between objects and subjects reaches a certain degree of stability. At that point, the contribution of the subject’s proactive disposition in defining the categories and their application to single experiences somehow conflates or is absorbed by the morphological representation of the objects or phenomena. This is a choice of linguistic/communicative economy, but it is not able to efface or supersede the semiotic relational gist of categories and meaning. On the contrary, taking the outcomes of the above communicative ellipses as ontological figurations of the way ‘things are’ amounts to nothing but a form of idolatric iconism that is doomed to escalate to an epistemic and communicative tyranny.

The cultural stability of the relational semiotic webs epitomized by categories and the a priori synthetic judgments they encapsulate can only induce us to lose sight of their probabilistic signification. Exchanging, and then passing off their inner probability as certainty obscures the proactive and enactive [42, 45] purport of categorization. The meaning they convey does not dwell in any static identity of their morphological structure, namely the set of elements composing their checklists; rather, it is coextensive with the dynamic outcomes resulting from the interpenetration between the semiotic means/ends texture that categories epitomize and the contextual environmental connections triggered by their use in the unfolding of human activities (both symbolic and pragmatic).

What is to be emphasized is that the environment should be understood, in turn, as a set of material and symbolic ingredients ready to play the role of signs in prompting and engendering the emersion of the relational framework hosting the enactive/proactive meaning of categorizations. This means that part and parcel of such a semiotic environment are also the semiotic oceanic landscapes enshrined within all human subjects. The genuine meaning conveyed by each category, therefore, stems from the interplay and interweaving among the signical means/ends scripts that each environmental situation, including the semiotic mnestic and cognitive apparatuses, includes. Such apparatuses are, in turn, collections of means/ends scripts that can be pragmatically presentified and enhanced in any situation so as to change the conditions of ‘signification’ of pre-existing categorical schemes. Each means can reveal itself as involved in different teleological/functional frameworks and, therefore, convey its experiential and effectual interaction and interpenetration. This blurring of semiotic scripts produces not only a polyfunctional signification of means but also the crossing of the pragmatic and symbolic paths allegedly epitomized by each end. The cumulative effect of such a dynamic is the remolding of the categorical spectrum of ends and the related means, which transmute in the components of the categorical checklists and then, meanings. In a sense, synonymous with this semiotic metamorphosis is an initial ‘gelling’ of new experiential spaces that will ultimately be the outcome of the proactive enaction of the signical apparatuses nestled in the mnestic stores of individuals’ minds. But what memory allows is the possibility to efficiently make present signs that are related to previously symbolized situations which have taken shape and place in other rooms of experiential time and space. ‘Elsewhereness’ and ‘remoteness’, and their signical components, are therefore full-fledged elements of present situations and are necessarily to be taken into account in determining its meaning as well as that of the categorical schemes used to deal with it. Each situation, taken in its signification, is thus to be seen also as a convergence of different spaces and times, both of which are understood as semantic frames. Semiotic and pragmatic transaction [38, 41], in other words, is the only way to grasp, and in a sense to make safe, the process of signification that in the past has led to the making of categories and cognitive/behavioral habits.

The question then, is whether contemporary legal systems, insofar as they are considered and managed in positivistic terms, are self-referentially able to maintain their preconditions of existence, validity and effectiveness by themselves.

## Law, Meaning and the Future

The thorny issue of the liberal narration and external/internal divide is that the figuration of what facts are is impossible without: (a) an aesthetic/cognitive activity and, thereby, (b) the involvement of a coefficient of indeterminacy in picking out from the relational experiential situations the elements to be considered as salient in shaping the means/ends dialectical dynamics from which meaning emerges. An individual’s selection of the salient elements, in turn, plays a dynamic role in the relational context (both symbolic and material) in which any human action enfolds. All this is confined at the moment of the creation of the initial (mythical) political social contract and, even before, of the semantic one coextensive with the emergence of the cognitive, communicative and social conditions that make possible its primeval bargaining. The initial semiotic spaces of freedom, conversely, should be called upon to reiterate their selective function according to the indetermined emergence of relational/situational frames, and so on. Any reactivation of the process should stop only in conjunction with the emergence of a third dimension, or thirdness, which, however, will constitute only an interlocutory pause along an endless and inherently open process.

The above aesthetic and free activity of proactive selection is genealogically intrinsic in any fact and in the production of its consequences, and therefore also in the unfurling of its meaning.^56^ Deciding what is the meaning of a ‘fact’, to some certain extent also implies a statement about what ‘it is.’ The issue of whether an individual disposition has only an internal significance or is outside the categorical/spatial frames corresponding to the behavioral content of the rights molded and conferred by law and political institutions, is destined to remain open, at least from a cognitive/aesthetic point of view. Moreover, it is not only an axiological issue but involves also matters of ‘facts’ and their semanticizations. Giving an absolute and ultimate answer to that question would be a mere apodictic assertion even because no political, social or institutional power or device is capable of maintaining the experiential conditions underlying the contingent significance of categorical spectrums. Rather than being tethered to the past and ingrained in a mythical cultural and political unity, as all the strains of positivism implicitly postulate, the semantic fabric embedded in each legal system is intimately prone toward the future. For the very same reason, the bundle of doctrines aiming at imposing literal, textualist or originalist readings of Constitutions are only rhetorical smokescreens in support of partisan interests.

Law is not a concept semantically folded within itself. In a slightly provocative fashion, it could be said that no ‘concept of law’ can ultimately be predicated with regard to legal experience.^57^ Law is rather a set of symbolic and material means, a *medium*, designed to concur to produce a future that includes itself. Its meaning unavoidably rests on what follows. And this because as a collection of signifiers, law continually pours its transformative and relational potentialities into the generation of a thirdness that blooms again and again on the verge of the renewing relations with the social environment and its plural, unforeseeable semiotic oceans. On that verge even the very measure of the validity of legal rules morphs and transmutes its actual meaning. Hence it is plausible to say that precisely this morphing is a result of the way in which facts and their semiotic weft undo the past semanticizations of words even and especially—it could be said—in the domain of legal experience.

The transformative perspective just outlined, however, does not embody a form of legal realism, nor even a constructivist cognitive and ethical approach. The ‘semantic past’ with which the legal discourse is imbued, far from being irrelevant or annihilated by the ‘supervening’, is to be taken as the pre-condition and an active ingredient of the future and the thirdness it enactively implies. But for this thirdness to be the outcome of an aware disposition, and not a mere occurrence that pops up in a given case, it requires that the hermeneutics of the legal system and its semantic components remain open to freedom and the creative, aesthetically driven activity inherent in any genuinely intelligent conduct.

The foregoing remarks about the relationships between facts, values/ends and meaning in the analysis of law as a systemic whole can be gauged on legal interpretation ordered to the application of legal principles and rules. This is the only way to grasp the implications of the secular epistemology—the real and historical matrix of both facts/values and is/ought divides—encapsulated in legal positivism and the scope of those implications with regard to the different branches of legal experience. In this regard, therefore, the initial question transforms into: What are the relationships between means and ends, the visible facticity and the semiotic invisible clouds, when judges need to qualify individuals’ behaviors in order to apply legal provisions?

In this regard, most recently, some voices have assumed a more nuanced and critically aware position.^58^ They propose a theory of semantic/normative *density*. Following this theoretical proposal, which in many ways recalls Hart’s solution to the problem of *hard cases* and the semantic vagueness of legal rules, each legal system self-regulates the intensity of its own hermeneutic and thereby systematic openness by pinpointing (more or less rigorously) the meaning of both the descriptive and the deontic parts of its rules. The problem with this approach, however, is that lawmakers are not able to use this ‘gradient of semantic density’ as—so to speak—a ‘potentiometer’ by virtue of which everybody can change at will how to light up or obscure the surrounding world. Especially in multilayered legal systems, ‘facts’, enunciated by the voices of people, claim for their semantic relevance with respect to basic values/ends, the same which (should) play the role of a yardstick for the validity of all the other rules. In determining the semantic density of the rules that it produces, lawmakers have to take that relevance into account; otherwise, those rules will be considered, for example, invalid (unconstitutional) precisely because they leave this or that respect of factual dimension of experience apart from its semantic scope. The argument that any positivist would raise at this point in all likelihood would sound more or less as follows: ‘but if the gradient of density is deemed invalid according to the basic values and principles of the legal system, it is in any case the positive law that determines its own validity scope.’ And yet, the tendency to trace backwards the legitimation of the hermeneutical variations cannot rhetorically immunize the legal system from its indeterminacy and, above all, veil the circumstance that such indeterminacy gushes out from the semantic and relational dialectics that unremittingly intervenes in its relationship with ‘natural’ and other languages. Furthermore, and once again, an inter-linguistic dialectical and transformative interpenetration depends on the inability of law to avoid including within its rules, explicitly or implicitly, words and/or categories pertaining to the natural language and its inherent transformations. On the other hand, the categories of non-legal languages encapsulate values and ends that are the same ones that society signified in originally legitimizing the birth of the legal system itself. This circularity cannot be faced, or rather eluded, by saying that the semantic and logical unity of legal systems is based on social facts, [73, 195] social structure, conventions, culture, ethics, and so on. For, at least, social structure, public opinion, culture, and ethics are anything but monolithic, static, semantically fixed, etc., and it is precisely such inherent dynamics that challenge, from both outside and inside, the semantic rigidity of legal systems and, in the end, the values/facts, is/ought, divide. I think, by contrast, that it is along the semantic mobile edge between factual categories and deontic ones, exteriority and interiority, objective and subjective, law and experience, that the axes of legitimation and validity of each legal system take their shape. This means that they are relentlessly renewing and forwarding their being into the future and its global semiotic dimension, coherently in tune with experience and its changing, unpredictable metamorphoses. All this is not outside but in the middle of what legal systems’ validity and existence means. Drawing inspiration from Peircean semiosis, therefore, it could be recognized that authenticity coincides with a thirdness that germinates and flows along the ridge of the encounter between past and present / future signs. And, as such, is always exposed to revision, to regeneration, with the production of a retrospective effect that cannot be normalized in the omni-significance of any kind fundamental norm or of the norm of recognition and with their derivations. On the contrary, and just for this reason, that creative retrospectivity calls into question the inescapable responsibility inherent in the search for meaning which is coextensive with the very production of experience and its indeterminability. There is nothing of Hegel’s ‘Concept’ in this, nothing allegedly taken for granted in advance, but only a kind of infinite, and perhaps humanly unbearable, awareness of the uncertainty of whatever cognitive and/or axiological certainty might exist. In short, to speak as a practical and disenchanted lawyer, it entails that the certainty in law as well as in life cannot consist in anything other than a thoughtful, weighed, and responsibly creative management of uncertainty.

Any attempt to conceptualize once and for all legal experience and its dynamics is, at bottom, an attempt to immunize law from unpredictability. As long as the scientific legal—and not only—mission is thought of as an effort to discover comprehensive, although formalized, schemes to capture experience, a collection of objectifying and universalizing assumptions will be its implicit and inevitable by-product, which will kill freedom and beget cognitive blindness to, and indifference towards, one another. The widely analyzed result will be a double dictatorship affecting both the domain of values and those of facts; the way in which the world should be and, at the same time, the way the world ‘is.’

At this point, although it would take me too far off topic to analyze the implications of the epistemology of secularization for private law,^59^ it is worthwhile to emphasize that even the presumed icon of modern autonomy, namely contract law, has been invested and affected with the dichotomy internal/external, facts/values, intention/motive. Just a few referrals to well-known doctrines can confirm these brief clues. Consider, in this regard, the divide, widespread in civil law tradition, between cause and motives in contract law [181: p. 12 ff.],^60^ as well as the objectifying signification of the common law category of ‘consideration.’ On average, in civil law tradition the seclusion of the internal sphere is justified in terms of the safeguarding from the expectations of the ‘other part.’ From another angle, legal theory and case law call into play the principle of due diligence in carrying out ones’ own activity, especially in the economic field, by gauging it, in turn, on the standard of the “rational/reasonable individual.” Consequentially, this individual will be deemed liable for any negligently unintended legal consequences of her/his actions (including speech acts). Therefore, once the judge identifies the contractual type corresponding to the individual agreement reached by the parties in question, they shall be bound to respect even the implicit obligations (*naturalia negotii, implicit obligations*) made inherent in that contractual type by the legal system; and this, furthermore, will occur regardless of whether these obligations have been explicitly provided in the agreement.

In the common law tradition, conversely, the divide revolves around the contract as a subjective free promise and the objective rationales for the enforcement of contract obligations rooted in the evidence of an agreement, a factual intervention of consent and, thereby, the promisee’s expectations (…which makes neither apparent nor real intention concerning the binding significance of the promise essential to the formation of contract and neither apparent nor real intention concerning the binding significance of the promise).^61^ Even in this case, the dialectics between ‘internal’ and ‘external’ converts into a polarized opposition between free will and objective normativity, to the point of inducing some [5, 64] to announce the ‘death of the contract’ and the reduction of its enforcement remedies to tort law. Beyond the claim for freedom and the opposed need to protect the confident expectation engendered in the ‘other part,’ in both civil and common law traditions even the most radical paladins of freedom of contract implicitly rely upon the intent/motives distinction and the ‘facticization’ of intent in the objective evidence of words’ meaning and their ‘factual referents.’ In some way, the dispute on the scope of contractual freedom they engage never goes beyond the issue of whether the parties have been willing to do one thing or another; the meaning of both, however, has already undergone a process of semantic compartmentalization and objectification. Even freedom of contract seems thus enmeshed in dialectics between the external and the internal, normativity (both semantic and axiological) and will. The objective meaning of words and behaviors is, however, the *finisterrae* beyond which the realm of the subject’s self-determination is to enfold in on itself.

And yet, the objectivity of meanings nestled in contract dynamics is imbued with values the determinant function of which is simply culturally camouflaged and dissimulated in the process of semanticization. This means that freedom and its attitude to function as a propellant for the renewal of categorizations and their spatial/behavioral implications results in the petrification in past cognitive patterns embodied by the well-established factual categories. Freedom, in this situation, ends up boiling down to an *indifferent-to-Otherness disposition* almost mesmerized and magnetized towards the prosecution of fixed and reified aims and goods, as such already compartimentalized, and mapped according to the institutionally and historically heterodetermined geometry of rights. By contrast, the dynamics of contemporary global semiotics make so that multiple ‘elsewheres’ increasingly blur and remold the semantic-spatial *continuum* on which the apparent stability/objectivity of facts depends. Law does not belong to a deontic realm that is ontologically distinct and phenomenally immune to the transformation of the semiotic and relational warp underlying the world of facts. It is completely involved in these transformative dynamics. Such involvement occurs because facts are structurally composed by axiological ingredients that are continuous to cognitive patterns and are dynamically called into question concomitantly with the legal systems’ apical values. Insofar as each ‘fact’ *genetically* embodies axiological elements that are not alien nor ontologically distinct from legal values,^62^ its continuity prompts the redefinition of the semantic boundaries. These semantic adjustments, in turn, can produce redefinitions of the facts and their categorizations. This process continually redefines, in a circular fashion, the semantics of the validity indices of legal systems, condensing into the dialectic emersion of a thirdness coinciding with a new overall situation in which facts and values have new relational connections and meanings. The more the facticizing and objectivizing consequences of secular epistemology hamper an aware and socially widespread embodiment of the facts/value dynamics, the more democratic societies will remain bereft of the cognitive and semantic coordinates needed to face the transformation of their experiential pre-conditions—in Kantian terms, the (even tacitly) shared a priori syntheses which steers social activities.

In order to reckon with the metamorphosis of the boundary conditions for the effectiveness of democracies and their legal systems it is not enough to renew the *political* social contract [82: pos. 1971 ff.] and the horizon of shared values that makes it possible. What is needed is to go a little deeper and develop a cognitive propensity to refresh the semantic social contract underpinning the lexicon of public communication and the understanding of ‘facts.’ Without the cognitive openness required to remold this semantic social contract, the ‘objects’ of the world and their categorizations will continue to remain fixed, thereby encapsulating implicit ends, which, as such, will have, still, a hold on psychological reactions, but only to elicit the activation of ossified cognitive and behavioral habits. Hence, implicit ends will become reified in predefined semantic schemes and will be made unavailable to their translation in symbolic terms. Such translation, however, is in turn a necessary step to make people able to disaggregate/dis-compose the means epitomized by those ends and glimpse the possibility of updating the paths of their teleological conduct in order to be responsive to the changing contexts of experience.^63^ When that dis-composition is not performed, people have no insight on the semiotic and pragmatic relationships underlying ‘things’ and the ‘related categories,’ consequentially they fail to understand the threads between what precedes and what follows the proactive/transformative inclusion of themselves in the unfurling of experience.

By contrast, the shadow cast by the ‘objectivizing facticization’ of the judgment of things, behaviors and phenomena and the underlying means/ends relationships lead democratic conflicts to achieve existential possibilities and social goods already frozen in a kind of cryonizing semantic and pragmatic stillness. In this scenario, freedom itself will become compartmentalized and thus demoted in its meaning and expressions, to nothing but a mere epiphenomenon of a public sphere already definitively mapped for both its meanings and its behavioral paradigms. Under these conditions, freedom’s expressions will simply ‘externalize’, if anything, in giving rise to unavoidable struggles among reified desires and stiffened patterns of conduct, which, as such, will be understood and performed as unrelated scripts immanently coextensive to ‘things’ and ‘facts’ idolized as unmodifiable icons to be pragmatically embodied and venerated only as fetishes. The withering of freedom’s innovativeness and its pigeonholing in fixed behavioral and semantic patterns—which people, in the name of a pre-packaged autonomy, will be free to activate or not but without any effective power/right about its ‘how’—fatally transforms democracy into a marketplace for the buying and selling of consent. On the backdrop of such commoditized participation, the democratic motor and horizon will be predominantly the distribution of goods, the value and meaning of which will have been previously and tyrannically boxed by a neutral and impersonal mechanistic device: the competition for goals and goods defined exclusively by their monetary equivalent transmuted in a universal and formal yardstick void of any meaning whatsoever. In this sense, incidentally, today than more ever it is really difficult not to consider as prophetic Marx’s [119] interpretation of money as ‘pure form,’ as such capable of producing a cognitive alienation of the human mind from the experiential and living relationality inherent in the significance of goods ‘engendered’ by the capitalist free market economy.

In the opposite direction, realizing that ‘things’, ‘objects’, ‘facts’, etc. and the related meanings are emergences of dynamic semiotic relationships could promote a de-reification of the democratic debate. The achievement of such a cognitive turn could convey the transformation of democratic participation in an inclusive experience oriented to figure out and trace new relational networks [149], as well as to update the previous ones and make them responsive to the changing and ever more mobile/plastic spatial and temporal semiotic circuits.

## Concluding Remarks: Democracy and Facticization, or People’s Delusion About the Effectiveness of Democratic Processes

Democracy entails transformation as well openness to it; and yet, any genuine transformation involves semantic changes. Hence, democracy and semiosis *should* be figured as two sides of the same coin. Precisely for this reason I am not in tune with those who argue—though not without reason—that the challenge for ‘equal freedom’ cannot be mediated by law but, instead, is to be attempted and carried out outside the legal domain and in communicative circuits marked by less stringent constraints of semantic pertinence.^64^ According to this view, the corrosive and hermetic criticism soaring, for example, from Kafka’s short story entitled ‘*Before the Law*’—written in the early twentieth century—should be read as a denunciation thrown against law in and of itself because of the cognitive and axiological tyranny inherent in its modern declination—in other words, a kind of *ante-litteram* visionary post-modern position. I am inclined to suppose, instead, that Kafka, as well as even before him Hugo in his ‘Les Miserables,’^65^ was only indicting the misuses of both the mythization of modern reason and law’s authority. Moreover, I would like to suggest that there is no room for semiosis without the possibility of stirring up some change inside (and originating from *people’s* ‘inside’) public language, including its legal dimension. On the contrary, any effort to set up a fresh semantic social contract from outside the legal language while still being embedded with social practices, more or less bypassing its ‘factual’ existence, would turn into an attempt to cut off the relationality inherent in social experience and meaning. But, as such, it would undermine even the chance of contributing to the emersion of a more inclusive ‘third space’ of signification [120, 126]; or, to be more specific, a constellation of third spaces, which could result from a commitment to the implementation and dissemination of the translation/transaction practices in tune with the dynamics of current global social semiotics and the semio-pragmatic challenges it throws at the survival of democracy and, all in all, the same category of ‘human.’ If some sort of *democratic-semiosic* process can start, reiterate and reproduce, we have to learn how to manage categories and the ‘proactive exteriorization’ they project on experience with cognitive ‘modesty:’ which means keeping them always open to translational/transactional relationships with Otherness and any *dose* of novelty and indeterminacy that is, inescapably, always on the verge of gushing out from *inside* its freedom [141, 142: § 6.301; 92: p. 321 ff.].^66^

The alternative is a (more or less aware) surrendering to the tyranny of ‘objective facts.’ However, in this case, it must be emphasized that such a dictatorship would be nurtured also by a feeling of individual powerlessness to counter it or, alternatively, to do without it in order to achieve people’s subjective ordinary goals. Actually, all over the world most individuals seem to be overwhelmed by a pervading sentiment that the destiny of the majority of human beings is, at its base, an implication of something overdetermining their choices—more or less, this is the way their quasi-Hegelian-reasoning goes. Consequentially, they cannot be themselves without complying with the ‘existent’ and the set of ‘standardized truths’ it comprises. This short chain of consideration is enough to silence most of them. A deep lack of confidence in the possibility to efficaciously—and, absurdly, even reasonably—criticize the ‘existing state of things’ and rules (both cognitive and legal) underpinning it hollows out their feeling as citizens. The prospective inanity of any attempt to criticize and relativize those rules engenders, by and by, a habit of blind aprioristic deference to them, which spawns the above sense of the ineluctability of the ‘existent’, as unfair as it may appear. In many respects, again, this is the secret source, a sort of collective spell, for the passive acceptance of the rampant arrogance that despite proclamations of human freedom and respect for human dignity affects the quotidian experience of many individuals in democratic societies of our day. The growing of an alienated and perhaps sometimes slow-witted acquiescence to the conduct of those who act in defiance of the same rules that the majority silently abide by is a clear sign of the above widespread deference for the ‘existent.’ In this way, almost by virtue of a dialectical subversion, the ‘unquestionable’ force of the rules scatters the pathogens causing their own final collapse. And so, what is invalid, aberrantly and increasingly, disguises itself as valid; the high-handed individuals pass themselves off as the keepers of that misguided version of ‘law and order,’ while common people feel themselves more and more powerless to criticize the ‘de facto’ power and without any hope in the possibility of being heard. In such overall conditions, legal systems become a set of signs without signification just like monstrous Hegelian entities, but—what is worse—drained even of any dialectic movement. In short, nothing but a tautological, immediate coincidence of legitimacy and justice with what ‘de facto’ is. The same factual reality that paradoxically seems to be doomed, on the other hand, to evaporate because it is swept out by events caused by humans, and yet out of any human control. Which would be nothing but the icon of an utter alienation of law/legal systems from human beings and their (alleged) aptitude for intelligent thinking and conduct. To use Vico’s [184: p. 62] metaphor: ‘non leges, sed *monstra legum*’^67^ or, even worse, the ushers to unintended catastrophes.

But words, including legal words, are bearers of semiotic elements, defining nevertheless spaces of experience and embodying them in a proactive and enactive way. For this very simple reason the things that are made with words are not done only with words but presuppose the entire landscape of semi-pragmatic implications that remain in the shade but give content to the words themselves and their relative inferences.^68^ And, therefore, the change in the world of facts, that is, of the semiotic elements that compose it, will spawn the undoing of what words, according to the theory of performativity, would have done on their own. In this regard, it misses the mark to say that the law presupposes that the factual conditions and the socio-cultural extremes of their semanticization remain stable. If so, as observed from the outset of this essay, the law would not make sense since its effectiveness would presuppose the permanence of the world and the related meanings accompanying its formulation at the very moment in which it is enacted. On the contrary, the law is primarily aimed at grappling with the future, as any categorization activity does, in view of and including in it the unfolding of an alteration of a series of pre-extant conditions. Categorizing implies abstraction and thereby inherently involves evaluation which, to have any effect, must necessarily include an entropy coefficient. Otherwise, in a world that is always the same, such as biblical Eden, categorization would not make sense, it would be redundant or … it would only lead to stimuli to imagine something that does not correspond to what it is … because it might not fit into one or another category: no less than the difference between good and evil [161]. And yet, this difference does not simply relate to the oppositional/dialectical relationship corresponding to being/not being, positive/negative, assertion/negation, entity/opposite of entity, etc. This is the world postulated by the positivist conceptions of law.^69^ According to these theoretical views, the law can exist, while keeping fixed the social and semantic extremes coeval to the conception of its norms, because the world defined in those norms could be disobeyed in a dialectical but unilateral way: someone does not respect the norm… and that’s it. In short: to abide vs. not to abide by the law. This kind of opposition presupposes a semantically static world. In other words, law and society would be a corpse and its symmetrical opposite. By contrast, however, the abstraction contained in linguistic categories and legal norms attempts to project its grasp over and through a future made up of differences, not just oppositions; of pragmatic-semantic drifts, not only from disobedience and violations. The ontologization and eternalization of the present (in many cases a mythically postulated present) are, in my view, the fundamental mistake of the foundationalist and justificationist conceptions of law. As paradoxical as it may appear, they are based, albeit to varying degrees, on communitarian assumptions and end up being somewhat authoritarian, even if they try to camouflage this implicit outcome under rhetorical labels such as neutralism, epistemological apriorism, normativism, self-reflexive justificationism, and so on. On the other hand, the same related radicalism in presenting the distinction between ‘is’ and ‘ought’ seems to me, at least in some cases, only a gargantuan epistemic strategy to affirm that there is a world out there—almost always corresponding to that of the socially dominant subjects—made of objective ‘realities.’ But it is precisely the strenuous defense of this empirical objectivity, one that is genetically crammed instead with values and corresponds to nothing other than the result of the processes of objectification of subjectivity and intersubjectivity projecting themselves onto experience, that is both the real objective and the lucrative gain of the dualist theses on the is/ought question and an absolute non-consequentialism.^70^ An ‘income’ that symmetrically overturns against those who allegedly receive it, since the postulation of a domain of subjectivity incommensurable to facts becomes the bulwark to avoid any sincere examination of the implications and therefore the meaning of the various postulations, positions, and even unreflective desires; nevertheless, at these, for various, and sometimes unspeakable reasons, people ideologically, or merely on instinct, peck away. Such a disposition seems to be very distant from the practical wisdom genuinely pertinent to the distinction between facts and values insofar as some cultural and environmental conditions show a degree of stability. Just the opposite way, the intransigent and aprioristic postulation of values/facts and is/ought divides—when taken to the extreme—pave the way to not much more that an ideological attitude—in the best case, a kind of semiotic ideology—and its self-reinforcing loops, the ultimate outcome of which is a dulling refusal to be responsive to the world and its load of Otherness. ‘Self-destructive, even if unintentional, freedom’ is a kind of blasphemy for modern thought. And yet, is this *unmentionable* wording the one best suited to making visible the huge and paradoxical concrete danger lurking behind the highly reputed ontological and moral reasons given to support the incommensurability between facticity and normativity? We humans would have much to learn from the global semiotics of COVID-19, especially as regards our imaginaries and the alleged foundation they would find in aprioristic expressions of freedom but tragically, we seem only able to …

## Footnotes

1 See [32] and, more in general, the implications of the enactive approach: in this regard, see at least, [42]. For further bibliographical references to enactivism and its relevance to legal experience I refer to [161, 163]. A proposal geared at bridging enactivism and both Peircean and structuralist semiotics can be found in [136].

2 See [168, 169] and [21], just to give two examples. It would be even more interesting to consider the so-called action theories (respectively end-oriented; value-oriented-immanently or expressively, and reasons-based) and their relevance for the ‘entanglement’ approach to the relationships between facts and values—on this see [16] and there for the related bibliography. Nonetheless, even in these approaches, the respectively teleological, axiological or rational elements play the role of external standards with respect to the meaning of the action/fact (or better, the action-producing-facts) units. But this is precisely the moot point that makes me take issue with *action theories*. In my view, facts are epitomes that stems from the relational and transformative interplay between the ongoing human action and the environmental (or external) elements of experience. The ‘final fact’, taken in its morphological features, is the outcome of a dynamic and transactive/transformative interpenetration enactively taking its own space between the activity progressively carried out by human agents and the environmental factors. Precisely because of this transformative semiotics it seems to me inconclusive to assert that, as related to each action, theory facts are partially outside goals, values and normative reasons: just because the final facts are not the same as the supposedly original external/environmental factual situation. Moreover, it is to be stressed that external facts become signs as soon as they are attracted to the inside of the pro-active horizon of action, and attention, of the individual. Insofar as facts are to be signified in order to be meaningful and, therefore, noticeable, perceptible, knowable, interpretable, understandable, and so on, they ‘are’ only to the extent that, and according to the way in which, they result from the semiotic translational/transactional interplay with human ‘specious’ and creative attention (or intentionality).

3 In this regard, I refer to the distinction proposed by Dworkin between ‘concept’ and ‘conceptions’ [48: p. 92 ff.]. However, I think that there is no difference, if not cultural, among what is assumed, alternatively, as a concept and/or as a conception. But see, in this regard, Simondon’s reproach addressed to the cognitive ‘ingratitude’ that lies in the any interpretation of phenomena blind to the genealogical process and the related components that are epitomized in their morphological appearance: see [174] and, from an explicit enactive perspective, [40].

4 I refer, here, to [59] interesting diagrammatic approach to categorization and indirectly to the radical conception of categories traceable, even if under different coordinates, in both [98] and [28, 29].

5 In this regard, reference must be made to al-Jāḥiẓ and his research on inter-categoriality carried out in the Kitāb al-ḥayawān (Book of Animals). On categorization in al-Jāḥiẓ see [127, 159].

6 The referral is to [10–12].

7 On this see [16: nt. 18], and his referral to Sellars’s [170: p. 255] ‘myth of given’. But in this sense see also [14, 32–34, 38, 71, 187, 188: p. 146–47, 110, 111]. In this regard, consider our judgements and perceptions (which are however the result of unaware inferences) about the state of the world. Imagine yourself, for example, on some past occasion sitting on a plane that was taking off or had just begun its descent, in both cases navigating in the dark of night. On average, especially if you were in the tail of the plane, you surely would have seen the front of the plane as it was tilted upwards or, during the descent, downwards. From an optical point view, both these visions are impossible insofar as you have always remained sitting at a right angle to the floor of the plane. How is it possible that you saw an inclination? The answer is: in those conditions and without any external point of reference you are seeing ‘with your ears.’ Actually, what is producing that inclined vision is your brain, which combines (in accordance with adaptive hierarchical criteria) the stimuli conveyed by the eyes and the ears—more precisely, the labyrinth, the inclination determined by gravity in the internal liquid and the cerebral interpretation of this signal with regard to the proactive disposition to manage the barycenter of your body should you stand up and walk. The whole inferential and adaptive/interpretive process takes place in the time frame of 500 microseconds, that is, totally beneath your awareness. And yet, the experience that spurred the brain to elaborate this perceptual pattern dates back to your infancy and is anything but unconscious. Yes, you fell on the floor again and again before learning the importance of managing your bodily barycenter. And actually, if now you were to see in an optically correct way, and you stood and walked, you would surely fall on the floor of the plane as if you were still an infant. Your adaptive *eye/ear* vision encapsulates sets of biological-proactive values/ends, but magically you ‘see’, namely perceive, the plane immediately, without being aware of any teleological inference. Analogically, seeing the color black is the result of an interaction between our biological structure and the stimuli from the external world. That interaction takes place according to values/ends which regulate it and are the result of evolution. And besides, colors do not exist outside our brain. Bees, for example, do not see black, but are able to see ultra-violet light by virtue of a visual system that is completely different from ours and has developed through, and because of, their evolutive interplay with flowers, which, in turn, developed their structure and colors according to their need to be pollinated by bees and other insects. If the coextensivity between facts and values can be observed and grasped at a biological level, its role will be even more plastic and relevant in the symbolic domain as will its tendency to become ‘invisible’ and ‘conflate with the perceptive/factual morphological appearances of ‘things.’ Against the classical Humean remark concerning the is/ought question, in which one cannot derive merely from the color of his/her skin that someone should be enslaved, one could radically rebut that her/his blackness is also the result of an operative blending between factual stimuli and human biological values. In the same venue, interesting enactively inspired remarks can be found in [23].

8 See, in this sense, [91, 115, 138, 139, 190, 191]; from a partially different perspective [48, 73]; but see also Greimas’ [67] analysis of legal language and the recognition of its self-referentiality. On Kelsen’s approach to the relationship between law, its object and meaning, see [137] and there for an interesting comparison between Kelsen’s principle of imputation and Krikpe’s skeptical paradox [97] (the so called *Kripkenstein*) according to which it is impossible to determine if a course of action is relevant with regard to a *semantic rule* by means of a mere reference to the facts of a past practice. A paradox, in turn, triggered by a famous Wittgenstein’s phrase according to which: ‘no course of action could be determined by a rule, because every course of action can be made out to accord with the rule’ [189: § 201]. From an enactive perspective, however, the gaps between facts and rules, is and ought, external and internal, objective and subjective, norms and values lack their logical—to be cognitively intended in Dewey’s terms: [31, 33]—presupposition. And this because meaning does not lie in an abstract of a-priori and self-contained ‘past’ standard but rather in the future and, because of its inner dynamic relationality, it is unremittingly open to its own operational readjustment to be intended, in turn, as coincident with its genuine and actual authenticity. From this perspective, moreover, also the presuppositions of Endicott’s approach to legal vagueness as well as, from a symmetrical perspective, Greenberg’s analysis about the way in which *facts would make law* [65, 66] appear devoid of their grounding assumptions. On the enactivist approach to moral issues and the related interpretation of the value/facts dualism, see, most recently, [41] and, there, the referral to [174]. For further references about enactivism see [163].

9 As for the ‘epistemology of secularization’ as the source of value/fact and is/ought divides, see [162, 163].

10 In this sense, legal doctrine should consider more carefully than it has apparently done in recent years, the critical studies devoted to the ‘interpretation of facts’ in legal reasoning and activity by scholars adopting the hermeneutical approach: see, for example [53, 54, 74, 88, 99].

11 As for the notion of a ‘semantic social contract’ see above and, in any case, [68: p. 103].

12 [31, 92].

13 [62, 84–87, 101–104].

14 [42, 45]. About the possibility to combine pro-active and enactive approach, especially as regards categorization, see [9, 39, 134].

15 [12: p. 23].

16 This is an argument which runs in parallel—even if it does not completely coincide–-with Putnam’s observation according to which also cognitive schemas [147: p. 166, p. 135–178; 148: p. 169, p. 156–159] are axiologically shaped and the related values are not incommensurable with aesthetic and moral ones, see also [133: p. 95] and [135], and from an enactive and 4E perspective [19]. In the same sense, but from a wider angle, [32].

17 [167].

18 …somehow evoking Vico’s reading of both linguistic, institutional and legal historical discourses: see [24, 25, 80, 183, 184].

19 See [38]. From an enactive perspective, see, most recently [26, 27]. See also [41], who very efficaciously observe: ‘Knowing always entails a relation of engagement that generalizes the logic of participatory sense-making […] in that in order to sustain an encounter between knower and known they must deal with a paradoxical condition. Both knower and known must be themselves as well as ready to be changed by knowing. At its fundamental, engaged knowing requires a particular attitude to flourish, the attitude of letting-be; otherwise, it degrades. Limited knowing can either take the form of overdetermination, i.e., a knower who attempts to force the known into an obstinate epistemic frame, or it can take the form of underdetermination, i.e., disengagement, a “respect” for the known that forgoes any serious relation with it, letting-be degrading into letting-go.’.

20 For the category of ‘semiotic ideology’ see [89].

21 This entropy has to do with the axiological/teleological axis genetically and constitutively inherent in any categorization. This axis functions as a qualitative and processive means—very similar to horizons and their prompting effect on both action and cognition—that continuously redefines its scope through the means, subsequently transformed in denotative and connotative elements that are dialectically involved in its achievement. This implies that categories can be thought of as epitomes and pre-figurations of integrated experiential situations within which means and ends interpenetratively and reciprocally mold their semantic spectrum. From this perspective, the entropy inherent in categories has nothing to do with (*extravagant*) vagueness as intended, with specific regard to law, by [51, 52] or, insofar as it is tied to i*ncommensurate multidimensionality*, by [4]—just to cite two examples. And this simply because vagueness is not to be justified with regard to an end external to the semantic structure of the word under examination (in Endicott’s [52] example the evaluative standard ‘neglect’ related to the conduct of parents with the purpose of preventing harm to children). The relationship between the end and the legal word is dialectical and mutually determines the respective semantic scope. This is the reason why the end does not transmit its value to the semantic vagueness of the word but, quite the opposite, concurs to operatively and situationally make the means partake in the overall situation engendered through the activity ordered to the achievement of the end. For the same reason, the incommensurate multidimensionality of the features constituting the categorical spectrum underlying a word or an expression have nothing to do with the informational entropy genetically inherent in categories. The exact semantic and operational scope of those features, as well as their reciprocal relationships, can be calculated only when the experiential process is exhausted (something that virtually never happens). The meaning and the topographical position of each feature within the categorical cartography cannot be considered outside the dynamic of their reciprocal relationship and the relational ties that each of them has with the overall semiotic network in which every category is embedded, and with which it is intermingled. From this perspective, any word or definition can become extravagantly vague or require a creative and relational redetermination of the semantic scope of its denotative and connotative elements. Only a dead world, the only one to which analytical assumptions about meaning could be integrally applied, could justify the reading of vagueness and incommensurate multidimensionality which both [52] and [4] configure. A world—to put things more bluntly—in which meaning and its production live exclusively in the past. Something similar applies also to Hunt’s [83] argument because of the formalistically oppositional and semantically univocal, rather than relational and transformative, way in which is configured vagueness in his approach and the other ones he takes in account. The contrastive analysis of Endicott’s approach and its presuppositions can be carried out –just to give some hints—through the following texts: [18, 32, 41, 123].

22 Latour’s formulation is as follows [100: p. 19]: ‘Everything changes if the word mediation fills out a little in order to designate the action of mediators. Then the meaning is no longer simply transported by the medium but in part constituted, moved, recreated, modified, in short expressed and betrayed.’.

23 In this sense, see also [102, 103].

24 And besides, also with regard to the Berlin key, Latour observes [100: 19]: “It is because the social cannot be constructed with the social that it needs keys and locks.”.

25 In this sense, see [14, 32, 132, 140, 143].

26 The referral is to [131].

27 The referral is to [189: §§ 46 ff.].

28 The same observation can apply to the example, suggested by [61], regarding the judgments and sentences related to artifacts as ‘This is a good car’ or ‘This is a good hygrometer.’ It seems to be valid both as a factual statement and as an evaluative one. But this depends—at least, in my view, which does not coincide with Geach’s theoretical premises—precisely on the circumstance that as an artifact, the car (or the hygrometer) makes somehow explicit, or at least knowable, the semiotic and material elements involved both its construction and morphological features. The point is that whatever judgment stems from the enactive relationship between the mind/body unit and the world. Therefore, it is genetically selective, so that what ‘is’, is in any case an ‘emergence’ of a relational adaptive values/ends-driven process. But see also [56] and, on both Geach and Foot about the relationship between ‘good’ and ‘contextualizing reason underlying its determination,’ [116].

29 …which I tie back to ‘legal chorology’: in this regard see [156, 157, 161, 164]. See also [146].

30 The main referral is to [109] theory of systems and [178] attempt to use it as an explanatory device to analyze and, in a sense, foresee the development of a planetary legal system inspired to a global reflexive constitutionalism. Indirectly, however, the same could be said for the application to social life of recipes, more or less inspired by post-modernism, that entrust the *progress* of the world and the ongoing unfolding of its inner relational dynamics to a de-subjectivized agency.

31 See, in this sense, the *pensive* and almost prophetic considerations proposed by Dewey in [35].

32 Aristotle, Parts of Animals, I. i. 639b–640b.

33 In this sense, Dewey’s remarks [31] prove to be prophetic even today and—to be blunt—completely overlooked, if not even neglected, by the same legal scholars that define themselves as inspired by pragmatism.

34 See [73, 91] and all their respective epigones.

35 I think that this shows clearly from the contrastive analysis of both Hart and Dworkin’s approaches proffered by Hart in his [73: p. 251–253]. Hart, in his theory of ‘open texture/indeterminacy/incompleteness’ postulates that where law is unable to be certain because its prognoses fail to grasp experience, there law must give way to ‘discretion’, namely creative activity to be carried out by judges. On the contrary, Dworkin assumes that the controversial issues surrounding the rules to be applied to cases must always be faced by means of a justificatory argumentative process that adjusts facts and rules on the grounds of the predetermined/pre-stated principles and their moral content. In Dworkin’s view, the system can always ‘engender’ consistency by virtue of a rhetorical effort oriented to find a moral/rational justification that makes the meaning of a rule and its application consistent to a case that is otherwise controversial or apparently crammed with discrepancies and incompatible profiles (in this regard, see also [47–50] and the related thesis of ‘one right answer’, ‘objectivity in law,’ ‘uncertainty/indeterminacy’ divide, the ‘constructivist approach to interpretation’, and ‘unity in value’). Both the above authors—as observed above—show a somehow misoneist view of law and its function. Legal provisions are not ordered—in a typical exasperated modernist way—to cage the future within their semantic and deontic schemas. And it is not so if only because this is not the function, and the aspiration, encapsulated in categorization. Categories are not a picture of reality or experience. Even if more than mere instruments for achieving external or heteroctonous ends, categories and representations are ‘a piece’ of experience dynamically and proactively integrated in its unfolding. They take shape along a dynamic ongoing process with respect to which they are only interlocutory co-elements. As such categories and representations are means to carry on action and implicitly include both the indeterminacy of the surrounding experiential environment with which they interact and its change. Their semantic variability, in other words, is part and parcel of their authenticity. In the same vein, law and its provisions try to capture and guide future experience implicitly and intentionally—metaphorically assuming that law per se has an intention—encapsulating in its meaning a certain degree of semantic and pragmatic entropy. This is the flip side of any attempt—which is, inter alia the nub of categorization—to use past experience and activity signification in order to orient the future moves. Hart’s assertion that when ‘fate’ makes legal rules controversial because of the appearance of something ‘unpredictable’ fails to grasp, in my understanding, a crucial aspect of legal experience and its internal ties with the rhythm of both signification and categorization. On the other hand, Dworkin and his argumentative coherentism are prone to gauge the search for legal meaning on a pre-existing and pre-woven tapestry of principles. In this way, the future is always legitimized and, in sense’ engulfed by the past, which is assumed as its yardstick of legitimacy. The argumentative endeavors, therefore, are designed—and even more ‘imagined’—to make the overall legal system fold in on itself and, ultimately, reaffirm a kind of pre-guaranteed identity with itself. Dworkin’s interpretive creativity, all in all, is a device of ‘repetition’, namely a movement that always re-states the same semantic script and semantic/pragmatic choreography. The changes, in Dworkin’s virtuous/moral universe, are only a façade: what changes are the implementations but not the concepts. By contrast, the inter-active and pro-active generating and unfolding of categorization as well as evaluation dwells within the interpenetration of what is experientially new and unpredictable and what is categorically and representationally forged by human knowledge and memory: and this precisely to (try to) give a compass for use in facing the adaptive challenges rising from the unwinding of experience. The genuine and authentic meaning of categorizations and moral/deontic statements is to be found, thus, on the dynamic edge between past and future that molds the signification of present experience. The overall semantic and axiological coherence of the legal system—as well as any linguistic or informational system—reconstructs and, simultaneously, renews itself originating at that edge. To put it more efficaciously, it is not the (meaning of the) present experience and its legality that is a consequence of the past but, quite the contrary, it is the (meaning of the) past that is a consequence of the future through the mediation of the present. And this, outside and beyond any Hegelian reassurance or guarantee rooted in the all-encompassing grip of Reason or Concept, constitutes a retrospective attitude of knowledge/interpretation that pivots on responsibility, responsive freedom, creativity, and a consistent dose of modesty self-reflexively gauged on the merely probabilistic and action-orienting ‘power’ of human cognition. In my view, semiotic openness, doubt, and genuine polyphonic participation should be the ingredients of the unremitting self-making of law through its own discursive elements; this is in sharp contrast to a law whose as-hypostatic-as-oneiric certainty makes it impossible to grasp these same elements, leading it to become fatally despotic when rhetorically invoked to hide, rather than solve, the inconsistencies germinating from experience.

36 In [73: p. 259] Hart defends his idea ‘that the courts exercise a genuine though interstitial law-making power or discretion in those cases where the existing explicit law fails to dictate a decision’; but this position is not very different from that which Kelsen argues, despite Hart’s dismissal of the *Stufenbau* with the epithet, ‘sky of formalists.’ And yet, who authorizes judges to exercise their discretionary decisions if not the same rules supporting their judiciary powers? Or, instead, in the legal system Hart imagines, are judges a sort of sacred caste endowed with some sort of sacred legitimation somehow recognized by people (namely, the legal subjects or citizens)? But this kind recognition would function as a kind of alternative ‘rule of recognition’, as such competing against the other rule of recognition that gives legitimate authority and public binding force to the rules enacted by parliaments. Otherwise, anyone could define themselves a judge only because they applied official legal rules or interstitially filled the space of indeterminacy of legal language in deciding the so called ‘hard cases.’ In this regard, I can only say that such conclusions seem to me to make little sense.

37 In this sense it could be observed that desuetude is inherent in the positive and expressed law. Desuetude partakes in the life of legal rules not only as regards the interpretive habits/consuetudes. It has to do with the cognitive plasticity of the phrastic parts of legal rules. In other words, desuetude concerns the means/ends relationships encapsulated in the categories comprising the descriptive/phrastic parts of legal rules. This is because the semiotically ‘implicit’ is part and parcel of the ‘explicit’ as well as the ends (or, at least, some of them); these are not external to the meaning/concept of word, objects and facts, but rather are intrinsically embedded in their formal structure or categorical checklist. If we fail to realize this, we cannot grasp the silent and radical ties between law’s certainty and the ineradicable diachronic, and thereby transformative, nature of legal experience.

38 Incidentally, this seems to me an intrinsic feature of many analytical approaches. It can also be found in the related conceptualization of intention as, for example, in [3]. Without dwelling on this important work, it is however relevant for the purpose of this essay, as her understanding of intention postulates an absolute, immediate and transparent correspondence between any individual’s intention and her/his understanding of its meaning. I think that it would be sufficient to think of how many times in our lives we ask ourselves ‘Do I want what I tell myself I want?’ to glimpse the relational implicativeness underlying any end in view, and its understanding. Something that could be summarized also by observing that representations are only a pro-active portion of experience and what we determine to be ‘meaningful.’ Against Anscombe’s anti-consequentialism, I merely contend that ‘implications’ are not ‘material/external consequences’, and that even these are semiotic implications that relationally, and thereby both prospectively and retrospectively, co-generate the self-understanding of our ends and intentions [33, 176]. It is no coincidence if the corollary that Anscombe connects to her correct statement that the end and meaning of intentional action are coextensive is that the intelligibility of Others’ behavior (but I add even of ours) requires that we project them within the spectrum of a limited range of predetermined ideas about what is to be considered as good. Alas, or instead fortunately for us, human life unfurls in a world without labels and pre-boxed, limited schemes previously guaranteed, as in a repertory, in their signification.

39 On Hart’s postulation of ‘the internal point of view’ and its ties with the overall external/internal issue, see below. For a polyphonic examination of that postulation, see Internal Point of View Symposium: downloadable at http://fordhamlawreview.org/symposiumcategory/internal-points-of-view-in-law-and-ethics/. Last Access 19 Sept 2020.

40 This is the reason I do not share a pivotal aspect of Dworkin’s criticism [46: 101 f.; 195: 229 ff.] of Hart’s conception of the Rule of recognition and its tendential conflation with ‘social facts.’ That the ‘legal’ can be exclusively thought of as ‘legal’, namely in legal terms and from an internal point of view, and that ‘legal validity’ cannot be derived from any from ‘social facts’ echoes the ‘naturalistic fallacy’ argument. But the is/ought divide, when applied to law (..and not only), postulates a class of value-free judgment that is only an invention of the epistemology of secularization. The circularity between facts and values in human experience, including the legal one, also undermines, on the other hand the distinction between principles and rules, as a—so to speak—pre-realist Ross had already shown in *Theorie der Recthsquellen* [165].

41 Regarding the claims for normativity, I would like to add just a few further remarks. The problem of normativists is that all their procedural standards for judging an assertion or a behavior (for example: reflexivity, rationality and autonomy, as [15] suggests) as morally/legally acceptable cannot avoid including semantic/substantive pre-assumptions that are engrained in specific worldviews—and beside even [57] ends up acknowledging this *substantive feature*. Despite the various attempts to relativize or reflexively get around the cultural constraints stemming from this *substantivity* of the premises of moral/normative rationality, it remains, in my view, an ineliminable flaw in any Kantian-oriented *justificationist* discourses and theorizations. What results from them is a way of translating Otherness that always looks one-sidedly oriented (for example, [70, 152]. There is no room for a genuine thirdness. The alleged universal morality engendered by such normative/discursive approaches simply does not envisage anything of the thirdness that can sprout from an open and aesthetically oriented process of semiotic translation able to *meet* Otherness along a line of symmetrical and creative activity of reciprocal self-transformation [32, 92, 140, 143].

42 In this regard, I consider Hart’s defense (in his postscript) against Dworkin’s criticism of his theory about the open texture of legal language and the discretionary power of judges when facing the so-called ‘hard cases’ to be really illuminating. More specifically I think it showcases how Dworkin’s ‘interpretive theory of law’ is a *misoneist* one. See [73: p. 276]: ‘Dworkin makes the further accusation that judicial lawmaking is unjust and condemns it as a form of retrospective or ex post facto law-making which is, of course, commonly regarded as unjust. But the reason for regarding retrospective law-making as unjust is that it disappoints the justified expectations of those who, in acting, have relied on the assumption that the legal consequences of their acts will be determined by the known state of the law established at the time of their acts. This objection, however, even if it has force against a court’s retrospective change or overruling of clearly established law, seems quite irrelevant in hard cases since these are cases which the law has left incompletely regulated and where there is no known state of clear established law to justify expectations.’ Through his criticism Dworkin shows, *ex contrariis*, that the interpretive approach is not retrospective and this just because according to it law, whether enacted in the past or however extant in social conscience/discourse, already includes all the possible solutions. Interpretation, as flexible or not-absolute as it may be in its results—at least according to Dworkin—constructs solutions that rhetorically (‘logically’) and politically already inhabit the law. Any decision is valid and not unjust insofar as it reiterates and reaffirms a legitimacy that implicitly coincides with the origin (the social Grundnorm) of the legal system. To put it diversely, the development of the legal system is already contained in its generative source. In a sense, the unfolding of the legal system’s effects is always a semantic enfolding in on itself. As asserted earlier, this really seems a recipe equivalent to the Hegelian maxim ‘Real is Rational,’ but made even worse by the lack of any referral to dialectics (however much it ultimately proved to be a mere façade even in the Hegelian system). The rationality of Dworkin’s legal experience appears, therefore, utterly self-contained in its own premises, that is to say that beyond its discursivity it is an authoritative social device cognitively blind to Otherness.

43 See [158, 162] for some considerations about the *kirpan affair* and there for bibliographical references on this topic. And even when these approaches rationalize the judicial *revirement* (for example Dworkin’s [46] reading of *Brown v. Board of Education)*, they are presented as a kind of applicative re-constitution of the ‘true’ meaning of principles and then already pre-existing conditions of systematic coherence. However valuable Dworkin’s [46, 48, 50] moral commitment, the axiological objectivism inherent in his theory of moral justification of law and legal decisions together with the adherence to the social/facts divide do not succeed in immunizing them from the tendency, stemming from the epistemology of secularization, to ‘normalize’ Otherness and make indifferent its difference.

44 A clear illustration of this effect of these non-positivist justificationist approaches can be found in the implications that [175] draws from Dworkin’s interpretivism (specifically ‘nonhybrid interpretivism’) as regards the issues, respectively, of ‘why does institutional practice matter?’ and ‘disagreement about the grounds of law.’ And yet, more in general, what does not work is Dworkin’s statement that the disagreement between jurists concerns theoretical rather than practical aspects. In my view, this is where the main methodological limits of Dworkin’s [48: p. 45–46] approach lies. He thinks that the interpretative approach should focus on the words of the law rather than on the words that are used to describe the facts and the facts themselves. On the other hand, Dworkin overtly declares how he is interested almost exclusively in ‘matters of law’s signification’ and not the issues of facts [48: p. 11]. In his eyes the issues of ‘facts’ concern, therefore, only the ascertainment of what materially happened without any consideration as to the meaning of events, objects, actions that have allegedly taken place and even less which categorical paradigms are to be used in ‘understanding facts.’ The universe of facts and its semantic self-evidence is simply taken for granted. It is outside the law and assumed as if it were absolute. He does not even consider the possibility that the self-evidence of the factual world is grounded on a pre-existent semantic social contract. It is hard to determine if he is affected by a radical naivety or, instead, by a hermeneutical blindness. But, from this point of view, he is not very far from Hart and his assertions about (legal) language’s ‘open texture.’ The mistake consists in the idea that the law is unable to determine, on its own, what meaning to attribute to the phenomena it intends to regulate. Despite all his interpretative efforts, Dworkin seems to maintain the Kelsenian-Hartian assumption: namely, that law forges its facts. This is the reason why he ends up referring to concepts as opposed to conceptions and, above all, believes that the interpretative variations with respect to concepts depend on the interpreter's own values, as such external to the construction of the concept itself. According to this view, once the concept has been built, the values/ends are to be considered neutralized and/or *engulfed* in meaning. The concept, in turn, is hooked, as regards its objectivity, to a sort of predetermined consensus existing at the social level or in any case oriented to predetermined values at the juridical-social level—that is, the constitutional principles, which Dworkin considers as moral assertions. Even when in ‘Justice for Hedgehogs’ he ends up recognizing that the values and the related interpretative problems already come to the fore with reference to the formulation of the legal concepts underlying the words of the legislator, Dworkin engages a deeper interpretative elasticity but never stops referring to the moral principles existing at the social level and fixed at the constitutional level. In short: he opens the debate on which values to call into question in weighing the correctness of legislative choices but always starting from the words of the law. Conversely, he continues not to address the problem that the concepts themselves—but it would be more correct to say the categories—insofar as they are related to empirical elements and situation, are inherently and genetically constructed on the basis of values, and that law is sensitive to these variations since the facts also exist in a different universe of language. A universe of language to which law cannot avoid referring and with respect to which it must necessarily hetero-integrate itself. And this, at least, if the right thing is to produce practical effects by intersecting the world of experience, which is, precisely, an intertwining of universes of discourse and meaning. The main problem with Dworkin’s reading of the semantic issue—or, as he calls it, the interpretive attitude—has to do with the idea that disagreement can be genuine only if it holds a common referential/objective correspondence in the world of experience, or better the world out there. He thinks, along with the positivist legal philosophers he criticizes, that a genuine blurring between different universes of discourse is impossible and that they—and their authors—would be doomed to talk past one another. His position, in other words, assumes that disagreements can really arise only if the semantics arguments of two ‘contenders’ refer to something objective extant out there. He does not seem to consider that any ‘objective item’ is in any case involved in proactive and enactive schemes of actions that are made only of semiotic reticular projections and that these semiotic schemes are neither inside nor outside the subjects’ mind but rather lie dynamically in the relation between the mind/body units and the environment. What comes into conflict, and at the same time lies at the origin of conflicts, are the common semiotic threads of which the scheme of actions—as such, in turn, epitomized in our categorizations of objects and events—are made of.

45 A wonderful exposition of the reasons underlying the thesis of the conceptual connection between law and morals can be found in [15], who makes it emerge from a dialectical comparison with the various version of ‘inclusive positivism’ in tune with the Hart’s Postscript to ‘The Concept of Law.’.

46 Pettit [145] closely analyzes Hart’s quasi-mythical and genealogical reconstruction of the law’s birth. The emersion of the legal dimension is identified with societal access in the domain of Lexitania. This constitutes a stage of development following a kind of cultural migration from a social condition called Normitania, connoted by the existence of primary rules of conduct, more or less equivalent to the regularity/*normality* of a collection of shared behavioral attitudes evolved in an even earlier stage labeled Pre-normantia. The passage to Lexitania marks the emersion, in social consciousness, of the secondary rules sprouting out from the recognition of the obligatory meaning of the primary rules. According to Pettit, emphasizing this genealogical plot in Hart’s theory of law would help to give the reason for which people internalize the rules of the legal systems in which they are legal subjects. Once again, the explanation of law’s existence, validity and obligatoriness is traced back to the past, more precisely a past taken for granted especially in its ‘semantic substance.’.

47 The referral is to Hart’s narrative phylogeny of legal systems through the stage respectively labelled through the wordings ‘Rex I, Rex II and Rex III;’ [73: p. 52 ff., p. 91 ff.] a sequence which in my adoption of Pettit’s rendition [145] becomes Pre-normitania, Normitania and Lexitania.

48 See [73: p. 256 f.] where he picks up the idea that rules can be obeyed for many reasons but without any conviction or sharing of their content. He does not put any thought into the possibility that not sharing or having a different understanding of the rule’s enunciation can overlap or interpenetrate.

49 In one way or another, such an assertion is tantamount to a sort of existentialist attitude, which overtly flaunts what the most sophisticated theorists, among the positivists, imperativists and formalists, try instead to dissimulate: the foundering of their semiotically static and structuralist positivism in an apodictic and retrospective (appeal to) social axiological and—what is even more worrying—semantic effectiveness; if not even, at least today, in an unintended resulting communitarism. [1, 195]. What, in many respects, seems to mark a tragical passage from the skeptical reductionism inherent in Hobbes’ motto ‘*auctoritas, non veritas**, **facit legem*’ (it is authority and not truth that makes law) to its perverse transformation in the self-alienating belief that ‘*voluntas, non significatio**, **facit legem*’ (it is will and not meaning that makes law). But on the hallucinating and dulling consequences of such ‘perversion’ I will get back towards the end of the essay.

50 On this circular argument that would make safe the formal consistency of Hart’s positivism and its distance from Dworkin’s approach to the relationship between moral standards and legal ones, see Shapiro [172]. But about Dworkin’s criticism to Hart see above nt. 7. On the contrary, I think that no real progress with respect to the identification of the rule of recognition with a social ‘fact’ can be traced in the ‘conventionalist’ turn of Hart’s Postscript. Despite the attempt to add a ‘bit’ of reflexivity to its previous presentation of the ‘rule of recognition’, the tautological paradox of a pre-packaged solution to the issue of meaning, and validity, of law remains unaltered. On the other hand, a similar remark can also be applied to all the versions of ‘inclusive positivism.’ These theories consider the ‘rule of recognition’ as a convention or a set of conventions whose future effectiveness would seem to coincide with the same existence of their own conclusions and the factual persistence of their cooperative compliance with it from legal officials. This abstract account of legal experience would align with the ‘concept of law’, to be understood as a sort of ‘ideal-type’, which outlines the ‘nature of law’ and has nothing to do with either its content, or its conditions of existence [20, 21]. The inclusion of ‘moral features’ in the language of law would not alter the ‘positivist’ significance of the preliminary conditions of its existence. In short, the validity and the same thinkability of legal systems does not rest in the meaning and in the dialectical significance of values/ends. As for the various declinations of ‘inclusive positivism’ and an insightful criticism of them, see [15].

51 This alienated world makes one think of the Hartian socio-legal scenario as a ‘reality’ embodying—as oxymoronic it may sound—a completely ‘externalized internal point of view’ on Law. But the same remark applies, in my view, even and despite appearances to the softened and quasi-conventionalist approach proffered by Hart in his ‘Postscript’ to ‘The Concept of Law.’ [195]. On the other hand, the conventionalism of Hart's Postscript, as well as that of other positivists, relies upon Lewis' [105] theory of convention. However, these authors seem not to deal with the problem of salience, the weak point of Lewis' approach, which, for this very reason, does not seem to provide a persuasive explanation of how conventions arise and evolve. For an introductory overview on this topic see [154].

52 And yet the situation is not brighter with Dworkin’s and other allegedly morally inspired approaches. This second passage clarifies what I argued before: that is, the exteriority of the end with respect to meaning and categorization. Utterance is the very beginning. The components of the interpretative attitude are independent of each other. The interpretative attitude is always and only assumed when someone wants to change the semantic rules. But this is completely untrue. The ‘authentic meaning,’ the one corresponding to the allegedly Dworkinian concept, cannot be grasped irrespective of the ends. In short, the teleological element does not come into play only in *hard cases*. Supporting this means, in some way, falling back into Hart’s false implicit axiom: that is, that the semantic social contract has ended once and for all. The law and its interpretations change because the world of facts, to which the rules must be applied and which those same rules try to ‘conform, change.’ But ‘that world’ is the same on which norms are originally and genetically designed to project their deontic and, at the same time, prognostic grasp. The authentic meaning of a norm is a processive concept, such as the Latin active periphrastic (or the English present continuous). There is no concept already settled, of course. The lawmaker’s intention springs from the past but is never tautologically reflected in it. The law looks to the future: otherwise, it would make no sense—except in cases where it says of itself that it is not to be intended retroactively. And this not because past events cannot be semantically redefined from the top of the present, from what follows them (think of the activity of re-flection) but simply because the law wants to influence future behaviors. From a genuine relational and reflexive semiotic point of view the ‘Wittgenstein/Kripke paradoxical skepticism’ [97] about the possibility to assert that someone is following a rule formulated in the past makes little sense. This also applies to the paradox proposed by Hart [72: p. 193–94] with regard to the apparent retroactivity in the qualification of legally relevant facts because their description is never the real source of ‘responsibility’ but rather a suggestion towards the calculation, in a previously undeterminable way, the ascription of it. In my view, these are only apparent paradoxes if only because there are no facts that are ontologically independent from an ongoing enactive relationship between the subject and the environment (in this regard, especially as for criminal conduct I refer to [162]. Actually, it is only apparently paradoxical that, among the future behaviors that law envisages there may also be the obligation to read the past in a certain way and to act accordingly: judgments always do. They ascertain in order to produce further action, to generate consequences, exactly as the *constative categorizations*—to use Austin’s terminology [6], to which I refer [163]. Even judgments including mere ascertainments still produce consequences, directly on the psycho-cognitive level, and indirectly on the practical one (in any case). For all these reasons, some kind of reconstruction of meaning always takes place: because the meaning of words and their insertion in a normative sentence must always measure itself with what will come, with what will happen and with its many facets. A ‘legal word’ that did not include this degree of entropy would only be one aimed at regulating what happened a moment before its enactment; if not even, according to an ideal simultaneity, at the very time of its drafting/emanation. It would coincide with being. It would be totally useless. For this reason, the distinction that Dworkin proposes between the concept and the result of an interpretation that is functional for the purposes or consubstantial with change simply makes no sense. A certain degree of change is intrinsic to the authenticity of the meaning of any legal term and, in some way, of any attempt at categorization. To the same extent that 'things' exist—that is, they are grasped as such by the human mind—because they are categorized, one could legitimately conclude that ‘they do not exist’ since they are only components, building blocks for the development of the subsequent experience within which they will acquire new meanings that cannot be predetermined in the present [92]. Those new meanings, however, are an integral part of current categorizations and meanings: the basic signification of which consists precisely in orienting future action or thinking. They are never the ontological mirror of something that is and that our mind can grasp in its ontological stillness and essentiality. This presumed neutrality of knowledge, which is the other face of the way of understanding and betting on objectivity, is only an illusion. Human knowledge is always oriented. However, the universe of values/ends is reticular, multicentric, rhythmic in its manifestation, taking shape from moving and variable axes of salience. History and convention are never able to fix a content: an argument that applies both to empirical categories and to those relating to the ‘ought.’ That fixing implicitly always includes in itself a certain entropy coefficient. Otherwise, the categorization would have no meaning. In an exhausted, finite world, there is no need to categorize. In a sense, everything has already been. In such a world, what could the law serve? Actually, trying to think of a command expressed in the present of a social universe that has already happened would be useless, if not simply impossible. This is why the final statement proposed by Dworkin about courtesy, that 'value and content have become intertwined' [48: p. 47] does not make much sense: they are always and processively entangled. Law does not—as Hart and with him all analytics would like—infinitely reiterate a semantic universe that is already concluded, extant and definitive. On the contrary, it is designed to support the production of a social field on the basis of some development premises, some teleological and prognostic indications, destined to function as a means to reflexively develop human society.

53 Law is an artifact in se but also a meta-artifact, that is, an artifact that functions as a means to build/engender other artifacts. Of course, in this second order of ‘acticity,’ which is its life and its being in-action, it has to come to terms with the matter it is molding… and it will change its meaning and function in order to interplay with that matter. In this way, it will become able to produce a ‘finished artifact’ that will retrospectively encapsulate its signification of meta-artifact by renewing both it and its primeval nature of artifact.

54 For some suggestions in this sense, see, more recently, [118].

55 Values/ends and meanings conflate and cooperate in the process of signification. ‘Function’ is not something separable or external to the construction of categories or concepts; on the contrary, it inherently determines the elements of the categorical checklists through a semiotic process. For this same reason, I think that the attempt to develop a legal pragmatism [21, 193, 194], within which concepts and their stability (at least, to a certain degree) can be detached from functions and ends, completely misses the semiotic relational and dynamic kernel of pragmatism as well as Dewey’s instrumentalism. Simultaneously, these approaches tend to give an *immanentist*—as it were—version of pragmatism somehow related to what I call the rhetoric of ‘form of life’, which is in turn drawn from Wittgenstein’s [189]. This means that the stability of legal concepts and the moral ones included by law in its language would be assured by social practice, which includes, of course, already reified ends. From this it comes forth that ‘To understand a legal provision is to grasp the pattern of inferences that underlies how the law has been used and to be able to recognize a variety of scenarios in which the provision would or would not be exemplified’ [194: p. 473]. This mixture of pragmatism (which tries to draw its philosophical pragmatist pedigree from Quine and his holism, Sellars and Putnam) and conventionalism [105] rather than reckoning with the axiological aspects inherent in the categorization of factual situations, coextensive with the ineliminable creativity involved in the construction of the second premise of the judiciary syllogism [160, 161], simply ignores it. By contrast, it transforms the recognizability of the situations allegedly predefined at the conclusion of the hypothetical semantic convention into a logical premise for the exercise of judicial function and a condition of its validity (and, ultimately, of the overall legal system). In short, we have again Hart’s factual understanding of ‘rule of recognition’ and the related foundational retrospectivism repeated with some (rhetorical) variations.

56 See [19] with specific regard to 4E cognition.

57 On ‘concept’ and the epistemological danger inherent in the use of this term see [14].

58 See, in this sense, the interesting arguments proposed by [128: p. 117 f.; 129: p. 26 ff., p. 33 ff.; 130].

59 On the epistemology of secularization as the source of value/fact divide see above, specifically nt. 4, and the referral to my other works and the related bibliography.

60 I refer to this book [181] for further references on this topic.

61 See the *First* (1928) *and Second Restatement* (1981). As for common law tradition debates about motives and contracts see, in a huge literature, [13, 81, 108: 7 ff; 8, 17, 58, 94, 96, 106, 107, 192].

62 On the continuity between the so-called cognitive values, on one side, and both the aesthetical and ethical ones, on the other, see above, and however [32, 147: p. 86; 148: p. 156–159, p. 169; 121, 122*vs.* 117].

63 With regard to the activity of semiotic dis-composition as a fundamental passage in the possibility to translate Otherness, see Ricca [155, 156, 164].

64 See [82: pos. 1971 ff.] against, among others, [69].

65 In this regard, see the insightful remarks proposed by [182].

66 See [180] for an insightful enactively-inspired examination of the flaws that the absence of such openness to transaction/translation produces in moral perception of Otherness because of the unaware assumption in normative terms of the embodied relationships and schemes of sense-making underlying and epitomized by the conscious categorization of behavioral habits. The de-ossification of such normativity, achievable by bringing up to the conscience the axio-teleologically oriented processes at the origin of the extant embodied relational sense-making schemes, would allow defusing the paradox of moral misperception caused by an as pernicious as unaware ignorance/blindness [63] to Otherness. About epistemic oppression see, moreover, [43, 44, 55, 93]; with specific regard to the intercultural perspective see [95].

67 English translation: ‘Not the laws, but the monsters of the laws’.

68 For an analysis of the performativity of ‘constative’ categorizations, denotation and their spatial implications see [163].

69 This applies to both Kelsen and Hart, who deduce, or simply dissimulate, the ‘scandal’ of semantic uncertainty—of which they are both acutely even if uncomfortably aware—in the authoritative power of judges.

70 In this sense, the charge of reductionism traditionally raised against moral naturalism does not consider that taking in account the relationship between moral postulations and natural conditions presupposes a culturalization of nature and an operational naturalization of culture. The process of reciprocal interpenetration between culture and nature, the domain of subjectivity and the domain of objectivity, internal and external, is essential to sense-making in both moral and scientific knowledge. On one side it is certainly true that ‘values’ make sense insofar as their realization corresponds to factual situations marked by possibility, and therefore the eventuality of their impossibility, otherwise they would be completely engulfed and neutralized by the absorbing dimension of ‘being’, with which they would coincide. On the other side, their significance, at least in regulative ideal terms, entails that they can both potentially and operationally merge with the factual dimension by remolding and transforming it. Excluding this interpenetration would aprioristically transform the moral dimension in something secluded from life or into a psycho-pragmatic corral of alienation. In short, if it is correct to say that values do not coincide with ‘being’ as they constitutively imply a dialectic of ‘absence’, it would be almost inane to apodictically assert that, moreover in the name of freedom, they can exclude any possibility of ‘presence’ and ‘achievability’; and thereby, that they are heterologous to the needed reciprocal adjustment between subjective teleological projections and the *environmental means* to be involved in the process designed to actuate and implement them—at least, as a postulated horizon of experience. In this sense, from an enactive perspective, as such assuming the axiological/normative configuration of both natural cognition and moral knowledge as well as the interpenetrative dynamics and operational continuity between them, see [41].
